# The development of depressive symptoms in older adults from a network perspective in the English Longitudinal Study of Ageing

**DOI:** 10.1038/s41398-023-02659-0

**Published:** 2023-11-25

**Authors:** Pascal Schlechter, Tamsin J. Ford, Sharon A. S. Neufeld

**Affiliations:** University of Cambridge, Department of Psychiatry, Cambridge, England UK

**Keywords:** Human behaviour, Psychology

## Abstract

An increased understanding of the interrelations between depressive symptoms among older populations could help improve interventions. However, studies often use sum scores to understand depression in older populations, neglecting important symptom dynamics that can be elucidated in evolving depressive symptom networks. We computed Cross-Lagged Panel Network Models (CLPN) of depression symptoms in 11,391 adults from the English Longitudinal Study of Ageing. Adults aged 50 and above (mean age 65) were followed over 16 years throughout this nine-wave representative population study. Using the eight-item Center for Epidemiological Studies Depression Scale, we computed eight CLPNs covering each consecutive wave. Across waves, networks were consistent with respect to the strength of lagged associations (edge weights) and the degree of interrelationships among symptoms (centrality indices). *Everything was an effort* and *could not get going* displayed the strongest reciprocal cross-lagged associations across waves. These two symptoms and *loneliness* were core symptoms as reflected in strong incoming and outgoing connections. *Feeling depressed* was strongly predicted by other symptoms only (incoming but not strong outgoing connections were observed) and thus was not related to new symptom onset. *Restless sleep* had outgoing connections only and thus was a precursor to other depression symptoms. *Being happy* and *enjoying life* were the least central symptoms. This research underscores the relevance of somatic symptoms in evolving depression networks among older populations. Findings suggest the central symptoms from the present study (*everything was an effort, could not get going, loneliness*) may be potential key intervention targets to mitigate depression in older adults.

## Introduction

Depression contributes significantly to the global disease burden [1] and is common among people aged 50 years and above in Europe and North America, with meta-analytic lifetime prevalence estimates of 16.5% [2]. More recent meta-analyses of individuals aged 60 years and above indicate that the global prevalence of depression is 28.4% according to questionnaire cut-offs [3] and 13.3% for a diagnosis of major depression [4]. However, depression prevalence is heterogenous across studies, countries, and age groups [2, 3, 5, 6]. In older populations, depression often remains unrecognized, with undertreatment leading to sustained impairment [7]. Under-recognition may be attributable to a substantial variability in the presentation and manifestation of depressive symptoms across the lifespan [8]. In older adults, depression often presents with more somatic symptoms than in younger populations [6, 9], yet current diagnostic systems conceptualize depression as a unitary, unchanging construct over the lifespan [10, 11]. To date, depression in older populations has been predominated by studies of cumulative sum scores [12], which can hide false assumptions that symptoms equally contribute to an underlying depression construct [13]. However, individual depressive symptoms have displayed differential associations with risk factors [14], comorbidity [15], and levels of impairment [16].

To elucidate the inter-relationship of depression symptoms, the network approach has been applied across different ages and populations [17, 18]. Instead of construing depression as a common factor with interchangeable indicators of disorder, the network approach defines mental disorders as a casual system of mutually interacting symptoms [19]. Analysis is focused on these symptoms; their importance (i.e., centrality) and interrelations can be examined empirically [20]. Core symptoms can be tested for clinical relevance in intervention studies [21, 22].

To date, network analyses have provided insight into the relative importance of different depressive symptoms across various populations [23], but network studies of depression in older populations are scarce. The few available studies in adults ages 50 and above point to the centrality of the symptom *depressed mood* [24–28]. However, these studies were cross-sectional or, despite being longitudinal, did not identify the directionality of symptoms. To test whether symptom relationships are temporal, network analyses must be conducted across a series of longitudinal models. This discerns whether some symptoms precede other symptoms, a central claim of network analysis. For instance, sleep problems may lead to concentration problems, which, in turn, could intensify feelings of anhedonia [19]. Identifying precursor symptoms has important implications as these symptoms can be targeted to stop future symptom activation. Temporal patterns among depressive symptoms were discerned in one longitudinal study of adults ages 50 and above where *feeling sad* and *depressed mood* were central symptoms which were predicted by many other prior symptoms [28]. However, this study aggregated data across waves and thus it remains unclear whether specific symptoms have a different impact at different stages during the ageing process [28].

Given the above limitations, a comprehensive examination of depressive symptom networks over time in older populations is warranted. This can advance knowledge of depression in older adults in two ways. First, it may reveal the existence of consistent network structures across different time periods, which could help to identify robust and replicable effects. This would provide crucial insight as network results are not always perfectly replicable both cross-sectionally and over time [17, 29, 30]. Second, this work may highlight unique network patterns that emerge across specific time points, particularly in instances where significant developmental events occur, such as retirement, shifts in social roles, or widowhood [6]. If consistent symptom patterns emerge over time, this may inform broad prevention programs, but if specific symptom constellations are limited to certain ages [8], this may inform more tailored approaches [6].

Specifically, it is important to discern the role of the hallmark symptom *depressed mood* across time, as this is one of two key symptoms required for depression diagnoses [10, 11]. However, longitudinal network analysis findings suggest that at the point of experiencing depressed mood many other symptoms are likely to have already been activated [28, 31], and thus will have already been causing functional impairment. Accordingly, the precursor symptoms to depressed mood may have relevance as warning signs of potential activation of diagnosable depression [32].

The role of loneliness in older adult depression networks is also understudied. While not a core symptom in standardized depression diagnoses [10, 11], older adults are often socially isolated and may be affected by separation, bereavement, and widowhood [6]. Increased loneliness in older adults has been associated with greater depressive symptom development, and conversely more depressive symptoms can reinforce social withdrawal and isolation [33]. Given the significance of loneliness among older adults [34], this symptom needs investigation within evolving depression networks.

Likewise, depression networks in older adulthood have not included somatic symptoms relating to fatigue (*could not get going anymore; everything was an effort*). Compared with younger cohorts, older adults are more likely to have reduced physical functioning due to chronic diseases or multiple comorbid health-related conditions [6], and from this may experience feelings of fatigue or increased burden. Likewise, disturbed sleep needs further study in older adults, as sleep is of high therapeutic relevance and influences depression treatment outcomes [35, 36]. In a longitudinal study, trouble sleeping had some outgoing connections but almost no incoming associations, indicating that this symptom may be implicated in the initiation of symptom cascades [28]. This could reflect a somatic pathway toward depression [8] with many outgoing unidirectional connections of somatic symptoms to subsequent mood-related symptoms [31]. Thus, understanding the role of these symptoms in longitudinal depression networks in older adults is key.

## Present study

The literature currently lacks a developmental perspective of how depressive symptoms operate in evolving longitudinal depression networks in older adults. Thus, we sought to investigate temporal depressive symptom constellations using data from the English Longitudinal Study of Ageing (ELSA), a nine-wave representative study of the English population above 50 years of age, with mean ages ranging from 65 to 76. Given the high and heterogenous levels of depression prevalence across age and particularly after age 50 [3, 5], this allows us to gauge whether the development of depressive symptoms is driven by different symptom dynamics across time in older adults. The present contribution focuses on the eight-item Centre for Epidemiologic Studies Depression Scale (CES-D-8), a psychometrically sound measure to assess depressed affect *(enjoyed life, felt depressed, happy, lonely*, and *felt sad*) and somatic complaints *(everything was an effort, sleep was restless*, and *I could not get going)* [37]. Using eight consecutive Cross-Lagged Panel Network Models (CLPN; 35), we aimed to disentangle how each of the CES-D-8 depressive symptoms may be predictive of, or predicted by, other depressive symptoms, and thus how central they are to depression in older adults. Based on the existing literature, we expected *feeling depressed* to emerge as one of the most central symptoms [28, 38]. Given the scarceness of longitudinal studies in older populations, we did not specify further a priori hypotheses.

## Methods

### Participants

The ELSA study focused on individuals aged 50 and above residing in private households in England [39]. The sample was drawn from participants of the Health Survey for England (HSE), which was boosted to ensure the representation of ethnic minorities. For ELSA, HSE households were excluded from the sampling frame if there was no adult aged 50 or older who had agreed to be contacted in the future. The remaining households provided the foundation for the ELSA Wave 1 sample that enrolled participants in 2002/2003. The study is representative of the population aged 50 years and older and consists of nine waves that took place once every two years. A multistage stratified probability sampling was used, and samples were refreshed over time to maintain representativeness. Core sample members are the 11,391 adults born on or before 29 February 1952 who initially took part in HSE. To allow for comparability of the networks, we focus on the core sample members with repeated-measures data in the present analyses. Demographic characteristics, assessment year, and participation rate for each ELSA wave are depicted in Table 1. Ethical approval was granted by the National Research Ethics Service (MREC/01/2/91). Participants provided informed consent. Data are openly available via the UK Data service. Our secondary data analysis was not preregistered (however, we note there are now valuable templates to preregister secondary data analysis [40]).

**Table 1:** Demographic characteristics for each wave.

	Wave 1 (2002/2003) (*N* = 11391)	Wave 2 (2004/2005) (*N* = 8780)	Wave 3 (2006/2007) (*N* = 7326)	Wave 4 (2008/2009) (*N* = 6623)	Wave 5 (2010/2011) (*N* = 6242)	Wave 6 (2012/2013) (*N* = 5659)	Wave 7 (2014/2015) (*N* = 4894)	Wave 8 (2016/2017) (*N* = 4219)	Wave 9 (2018/2019) *(N* = 3660)
Participation rate (%)^a^	–	77%	64%	59%	55%	50%	43%	37%	32%
Age (*SD*)	65.19 (10.23)	66.81 (9.77)	68.19 (9.55)	69.65 (9.08)	70.96 (8.66)	72.25 (8.26)	73.50 (7.74)	74.06 (6.62)	75.62 (6.36)
Gender									
Female	6205 (54%)	4830 (55%)	4181 (58%)	3708 (56%)	3500 (56%)	3181 (56%)	2767 (57%)	2384 (56%)	2092 (57%)
Male	5186 (46%)	3950 (45%)	3354 (42%)	2915 (44%)	2742 (44%)	2478 (44%)	2127 (43%)	1835 (44%)	1568 (43)
Ethnicity									
White	11065 (97%)	8586 (98%)	7382 (99%)	6489 (98%)	6102 (98%)	5527 (98%)	4784 (98%)	4118 (98%)	3573 (98%)
Non-White	320 (3%)	194 (2%)	153 (1%)	134 (2%)	140 (2%)	132 (2%)	110 (2%)	101 (2%)	87 (2%)
Education									
Less than secondary	4878 (43%)	3475 (40%)	2856 (39%)	2404 (36%)	2179 (35%)	1917 (34%)	1544 (32%)	1255 (30%)	1025 (28%)
Upper secondary	4256 (37%)	3481 (40%)	3055 (42%)	2746 (41%)	2640 (42%)	2434 (43%)	2205 (45%)	1935 (46%)	1713 (46%)
Tertiary	1257 (20%)	1057 (20%)	955 (19%)	902 (23%)	884 (23%)	828 (23%)	738 (23%)	677 (24%)	608 (26%)
Retirement									
Yes	5774 (51%)	4776 (54%)	4488 (61%)	4385 (73%)	4485 (72%)	4447 (79%)	4043 (81%)	3645 (86%)	3228 (88%)
Employed									
Yes	3607 (32%)	2516 (29%)	1976 (27%)	1467 (22%)	1107 (18%)	771 (14%)	534 (11%)	340 (8%)	233 (7%)
Marital Status									
Married	7570 (66%)	5882 (66%)	5054 (69%)	4511 (68%)	4342 (70%)	3987 (70%)	3501 (72%)	3053 (72%)	2666 (73%)
Divorced	857 (5%)	687 (7%)	613 (8%)	535 (8%)	510 (8%)	468 (8%)	406 (8%)	364 (9%)	327 (9%)
Never married	580 (5%)	422 (4%)	361 (5%)	320 (5%)	275 (4%)	245 (4%)	205 (4%)	171 (5%)	153 (4%)
Partnered	283 (2%)	236 (3%)	198 (3%)	183 (3%)	173 (3%)	163 (3%)	154 (3%)	143 (3%)	133 (4%)
Separated	140 (1%)	103 (1%)	89 (1%)	76 (1%)	76 (1%)	70 (1%)	58 (1%)	51 (1%)	42 (1%)
Widowed	1959 (17%)	1448 (16%)	1219 (17%)	977 (15%)	865 (14%)	725 (13%)	569 (12%)	436 (10%)	338 (9%)
Psychiatric Diagnosis									
Yes	765 (7%)	741 (8%)	687 (9%)	661(10%)	653 (10%)	634 (11%)	576 (12%)	496 (12%)	429 (12%)

^a^relative to the baseline at wave 1.

### Measures

#### CES-D-8

The eight-item version of the CES-D was administered at all nine waves [37]. The CES-D-8 is a shortened version of the CES-D 20-item self-report questionnaire [41]. It displays similar psychometric properties as the longer version in different populations, including adults aged 50 and above in Ireland [42] and adults aged 70 and above in the United States [37]. The scale assesses depressive symptoms from the previous week (Table 2 for item wording). The CES-D-8 uses a dichotomous response format instead of the original four response options to reduce participant burden and confusion, but this change does not affect the scale’s psychometric properties [37]. The dichotomous response format yields scores ranging from 0 (no symptoms) to 8 (all symptoms). The items *happiness* and *enjoying life* were reverse coded. Evidence for both a two and a one-factorial solution has been reported, including strict longitudinal invariance for both [43]. Items cover symptoms of depressed affect and somatic complaints, which constitute the factors of the two-factor solution.

**Table 2:** Percentage of participants endorsing the dichotomous CES-D-8 items across waves, as well as sum scores, internal consistencies, and item-level skew and kurtosis.

During the past week indicate whether…	Wave 1 (*N* = 11391)	Wave 2 (*N* = 8780)	Wave 3 (*N* = 7326)	Wave 4 (*N* = 6623)	Wave 5 (*N* = 6242)	Wave 6 (*N* = 5659)	Wave 7 (*N* = 4894)	Wave 8 (*N* = 4219)	Wave 9 *(N* = 3660)
1 …you felt depressed?	17.92	16.47	15.06	14.49	14.42	12.38	11.76	12.24	11.65
2 …you felt everything you did was an effort?	23.97	22.70	21.23	19.55	21.36	19.27	20.17	20.73	20.61
3….your sleep was restless?	40.97	42.35	40.73	33.74	40.21	32.85	39.80	35.16	42.48
4….you were happy?	88.93	89.52	89.81	90.07	89.96	90.65	90.92	91.68	91.35
5….you felt lonely?	13.83	14.15	13.83	13.49	14.26	12.32	11.67	12.26	11.86
6….you enjoyed life?	90.26	90.14	90.60	90.72	90.15	90.73	91.49	92.46	91.47
7….you felt sad?	20.74	21.46	19.22	20.05	20.93	17.87	16.63	19.42	18.42
8….you could not get going?	22.01	21.35	21.63	20.31	22.32	19.43	20.84	20.85	20.73
Cronbach’s alpha, full scale (α)	0.92	0.91	0.92	0.92	0.92	0.92	0.91	0.90	0.90
Depressed affect (α)	0.91	0.91	0.92	0.91	0.90	0.90	0.90	0.88	0.90
Somatic complaints (α)	0.80	0.78	0.77	0.80	0.78	0.78	0.76	0.75	0.75
Omega total full scale (${{\rm{\omega }}}_{t})$	0.93	0.92	0.93	0.92	0.92	0.92	0.92	0.90	0.91
Depressed affect $({{\rm{\omega }}}_{t})$	0.91	0.91	0.92	0.91	0.90	0.90	0.91	0.88	0.90
Somatic complaints ${({\rm{\omega }}}_{t})$	0.82	0.80	0.80	0.82	0.80	0.80	0.79	0.79	0.78
Skewness/Kurtosis	Wave 1 (*N* = 11391)	Wave 2 (*N* = 8780)	Wave 3 (*N* = 7326)	Wave 4 (*N* = 6623)	Wave 5 (*N* = 6242)	Wave 6 (*N* = 5659)	Wave 7 (*N* = 4894)	Wave 8 (*N* = 4219)	Wave 9 *(N* = 3660)
1 …you felt depressed?	1.67/ 0.80	1.81/ 1.27	1.95/ 1.82	2.02/ 2.02	2.05/ 2.18	2.28/ 3.22	2.37/ 3.63	2.30/ 3.31	2.39/ 3.71
2 …you felt everything you did was an effort?	1.22/−0.51	1.30/−0.30	1.41/−0.02	1.54/ 0.36	1.40/−0.05	1.56/ 0.43	1.49/ 0.21	1.44/ 0.88	1.45/ 0.11
3….your sleep was restless?	0.37/−1.87	0.31/−1.90	0.38/−1.86	0.69/−1.53	0.40/−1.84	0.73/−1.47	0.42/−1.83	0.62/−1.61	0.30/−1.91
4….you were happy?	−2.48/ 4.16	−2.58/ 4.66	−2.63/ 4.92	−2.68/ 5.18	−2.66/ 5.07	−2.79/ 5.08	−2.85/ 6.11	−3.02/ 7.10	−2.94/ 6.65
5….you felt lonely?	2.09/ 2.39	2.06/ 2.23	2.10/ 2.39	2.14/ 2.57	2.04/ 2.18	2.29/ 3.26	2.39/ 3.70	2.30/ 3.29	2.36/ 3.57
6….you enjoyed life?	−2.72/ 5.37	−2.69/5.25	−2.78/ 5.74	−2.81/5.88	−2.69/ 5.25	−2.81/ 5.88	−2.97/ 6.83	−3.21/ 8.33	−2.97/ 6.81
7….you felt sad?	1.44/ 0.08	1.39/−0.07	1.56/ 0.44	1.50/ 0.24	1.43/ 0.04	1.68/ 0.81	1.79/ 1.21	1.55/ 0.39	1.63/ 0.65
8….you could not get going?	1.35/−0.18	1.40/−0.05	1.38/−0.10	1.48/ 0.18	1.33/−0.23	1.54/ 0.39	1.44/ 0.06	1.43/ 0.06	1.44/ 0.08

#### Missingness

There was a high level of attrition among core sample members who participated in wave one (*N* = 11,391). Missingness rates peaked in wave nine at 70% (*N* = 3660). Little’s test for missingness [44] revealed that data were not missing completely at random, *p* < 0.001. Instead, sample characteristics predicted missingess: being non-white (vs. white), older, unmarried (vs. married), having a lower level of education, and greater depression severity, all *p* < 0.001 (consistent with [45]). This supports the assumption that data were missing depending on observed variables and are thus likely to be Missing at Random (MAR). Under this assumption, multiple imputation using these observed variables can help estimate unbiased parameters [46]. Given that network analysis is currently not compatible with multiple imputation methods, we only used one imputed dataset as recommended [47]. We imputed data by chained equations using the MICE package in R [48]. Given the binary responses, logistic regression was used for imputation [46]. In our imputation models, we included the variables associated with missingness as auxiliary variables.

#### Analysis strategy

We performed all analyses in R [49]. To examine temporal effects, we used CLPN modelling [47]. CLPN provides an ideal framework for our research question. It allows analysis of relationships between symptoms as directed paths over time in panel data with a few discrete measurement occasions (for an overview of other network modelling approaches and their application see [50]). Compared to cross-sectional networks, the directed paths in CLPNs indicate the symptom flow from one measurement occasion to a subsequent measurement occasion. These directed paths represent the shared variation between a symptom at time t and another symptom (either the same or different) at time t + 1, while accounting for all other symptoms at time t. We calculated the networks for consecutive timepoints, resulting in 8 network models (i.e., T1 → T2, T2 → T3, etc.). This way, we could compare the predictions of symptoms across different networks. Owing to the dichotomous response format of the CES-D-8, logistic regression models were used to compute autoregressive and cross-lagged coefficients. In *autoregressive pathways*, a symptom at one timepoint predicts itself at the next timepoint, adjusting for all other symptoms at the first timepoint. In the *cross-lagged pathways*, a symptom at one timepoint predicts a different symptom at the next timepoint, adjusting for all other symptoms at the first timepoint. We transformed the coefficients of the logistic regressions (i.e., edge weights) from log odds to odds ratios (ORs). This allows the interpretation of edge weights greater than 1 as a positive relationship and edge weights below 1 as a negative relationship; edge weights of 1 have no relationship. To estimate the regression coefficients, a penalized maximum likelihood with a lasso penalization was used [47]. A 10-fold cross-validation tuning parameter was applied so that small regression coefficients were set to zero [47]. We estimated the CLPN regressions using the glmnet package [51]. Gender and ethnicity were included as covariates as these were critical covariates in previous ELSA depression studies [52]. In addition, we ran cross-sectional network analysis on the nine-waves using the Ising model, which is able to handle binary data to compare the cross-sectional associations with the temporal associations [53].

We calculated the cross-lagged in-expected-influence and out-expected-influence as centrality indices [47]. In-expected-influence quantifies the degree to which each symptom is predicted by other symptoms in the network (the sum of the values of incoming edges associated with a symptom). Out-expected-influence describes the degree to which each symptom predicts other symptoms in the network (the sum of the values of outgoing edges associated with a symptom). To compare the networks across time, we compared the number of replicated edges across networks. In addition, we calculated the correlation among edge weights and centrality indices across networks [47].

The accuracy of edge weights was estimated by computing 95% confidence intervals (CIs) around each edge weight with nonparametric bootstrapping with 1000 iterations. To estimate the stability of our results, we used case-drop bootstrapping (correlating the centrality indices from the entire sample with centrality indices estimates on subsets of the sample) using the *bootnet* package with 1000 iterations [54]. In addition, we used the *edge weight difference test* and *centrality difference test* to pinpoint whether edges and centrality indices differ from each other significantly. The former test quantifies whether specific cross-lagged symptom connections (i.e., edges) are more important than other cross-lagged symptom connections. Likewise, the latter test quantifies whether some symptoms are more important (i.e., central) in the networks than other symptoms.

## Results

Descriptive item-level statistics can be found in Table 2. Overall, *restless sleep* had the highest endorsement across all nine waves (following reverse coding of *being happy* and *enjoying life*). Endorsement of *feeling depressed* decreased over time and endorsement of *enjoying life* and *happiness* increased over time (*p* < 0.001), while the other items showed inconsistent fluctuations (Table 2). The networks exhibited good accuracy, as evidenced by edge weights with small to moderate bootstrapped CIs (supplemental Figs. s4-s11) [54]. All eight networks showed strong stability of the centrality measures as evidenced by case-drop bootstrapping results (supplemental Figs. s12-s19).

### Longitudinal network comparisons

Networks were consistent over time. We observed strong correlations between cross-symptom coefficients in the networks (i.e., edge weight correlations, *r* = 0.73 – 0.98). Correlations were also strong across networks for both how much each symptom predicted other symptoms in the network and how much each symptom was predicted by other symptoms (out-expected-influence *r* = 0.55 – 0.92 and in-expected-influence 0.88 – 0.98, respectively). Further, consistency in the association of symptoms over time was demonstrated by cross-network replication of 80.3% – 91.7% of the edges in the networks. The cross-sectional networks of the nine waves revealed converging patterns of association compared to the temporal networks (Supplemental Figs. s2 and s3).

#### Autoregressive and cross-lagged edges

**Fig. 1:**
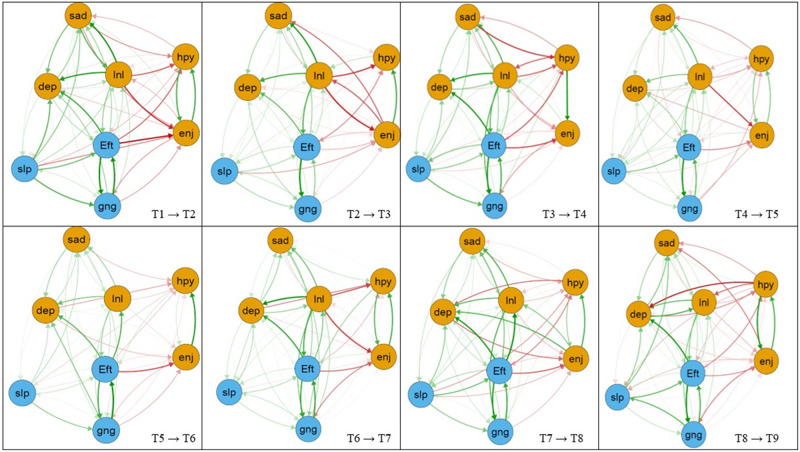
The cross-lagged panel networks for consecutive time-points. *Note*. Dep = felt depressed, Eft = everything you did was an effort, slp = restless sleep, hyp = happy, lnl = lonely, enj = enjoyed life, sad = felt sad, gng = could not get going. Arrows represent unique longitudinal relationships. Green edges indicate positive relationships; red edges indicate negative relationships (note that there are negative relationships as *happy* and *enjoyed life* were coded in the opposite direction as the other items). Edge thickness displays the relationship strength. Autoregressive edges and covariates were excluded to enhance visual interpretation. Yellow nodes represent symptoms of “depressed affect”. Blue nodes represent symptoms of “somatic complaints”. We used these factors according to the two-factor solution of the CES-D-8. Non-significant cross-lagged paths are excluded. All networks were visualized with an average layout using the qgraph package [77]. Nodes represent symptoms and arrows represent estimates of cross-lagged effects. The color of the arrows represents the directionality of the effect (green = positive effect, red = negative effect). Thicker arrows indicate stronger effects; non-significant cross-lagged paths were excluded. Nodes that cluster more strongly are placed together in the graph [78]. For better visual interpretation, nodes were colored according to the two-factorial solution of the CES-D-8 scale (depressed affect & somatic complaints). The underlying algorithm visualizes line thickness as a function of the strongest paths. For a better visual interpretation of the cross-lagged paths, we plotted networks in which only the cross-lagged effects are shown [47]. Bivariate connections among all symptoms over time (i.e., edge lists) are found in Supplement 2.

Figure 1 shows the eight CLPN models for all consecutive timepoints, depicting only coefficients where a symptom predicted another symptom (cross-lagged effects). When looking at the effects of symptoms on themselves over time (autoregressive pathways), *loneliness* exhibited the strongest autoregressive effect across all nine waves (OR: 6.33–9.33, Supplemental Material 3, Supplemental Figs. 1), with *restless sleep* (OR: 4.18–6.15) generally demonstrating the second highest autoregressive effect.

In four out the eight CLPN models, *everything was an effort* → *could not get going* displayed the strongest cross-lagged edges (OR: 1.80–2.33). In the other networks, this cross-lagged edge was at least among the top three strongest edges (OR: 1.94–2.10). Also, across waves these symptoms displayed significant cross-lagged edges in the opposite direction (*could not get going* → *everything was an effort*; range OR: 1.51–0.2.32). Moreover, a significant cross-lagged edge was found for *everything was an effort* → *feeling depressed* in all networks (OR: 1.45–2.12). Another consistent cross-lagged edge (edge rank position 1–9 across networks) was *loneliness* → *feeling depressed* (range OR: 1.64–2.34). Also, *loneliness* → *sadness* (OR: 1.47–2.20) displayed significant edge connections across all waves expect wave 8 → 9 network (OR: 1.20). The edges *loneliness* → *everything was an effort* (range OR: 1.35–1.97) had significant connections in all networks apart from the wave 7 → 8 network (OR: 1.15). *Everything was an effort* → *loneliness* (range OR: 1.24–2.21) had significant connections for all waves. *Enjoying life → happiness* (range OR: 1.62–2.14) and *happiness* → *enjoying life* (range OR: 1.23–2.03) displayed significant connections across all waves apart from the wave 3 → 4 network and the wave 2 → 3 network (both OR: 1.00). Edge weights difference tests for all networks indicated that the aforementioned edges (apart from the exceptions) were significantly stronger (*p* < 0.05) than most other edges across waves (supplemental Figs. s20-s27).

#### Centrality

**Fig. 2:**
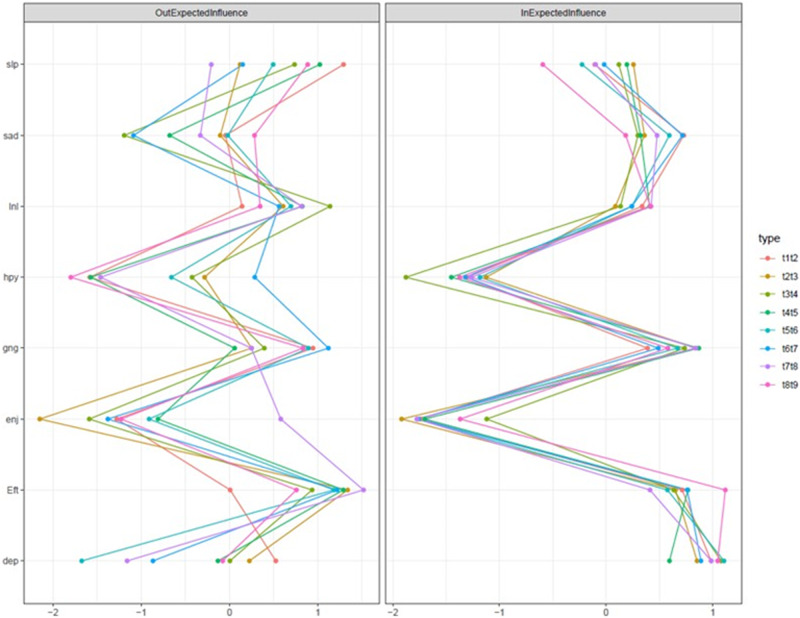
Symptom centrality estimates for the networks using z-values. *Note*. Greater values indicate greater centrality. Dep = felt depressed, Eft = everything you did was an effort, slp = restless sleep, hyp = happy, lnl = lonely, enj = enjoyed life, sad = felt sad, gng = could not get going. Type refers to waves used in each network model (t = timepoint).

Figure 2 depicts the standardized centrality measures. Across waves, *feeling depressed, everything was an effort, and could not get going* had the strongest incoming connections (i.e., in-expected-influence). As can be seen in the eight difference plots in the supplement (Figs. s28 – s35), these symptoms had significantly greater (*p* < .05) in-expected-influence compared with most other symptoms. The symptoms *loneliness* and *being sad* also showed relatively high in-expected-influence. The lowest in-expected-influence emerged for the two reverse coded symptoms *not being happy* and *not enjoying life*. They showed significantly lower in-expected-influence compared to the other symptoms.

For outgoing connections (i.e., the centrality measure out-expected influence), the symptoms showed more variability across waves. *Feeling depressed* showed relatively low values. *Everything was an effort, could not get going* and *loneliness* were consistently among the symptoms with the strongest out-expected-influence. Difference plots in supplemental Figs. s32 to s40 show that these symptoms had higher values than many symptoms across time. At some but not all waves *restless sleep* had a high out-expected-influence especially at the wave 1 → wave 2 network (Fig. 2). Apart from two networks, *happiness* and *enjoying life* had low out-expected-influence across waves and thus most symptoms displayed significantly higher values across waves (Figs. s36-s43).

#### Summary of key findings

The findings can be summarized as follows. The associations between symptoms remained consistent over time. *Everything was an effort* and *could not get going* displayed strong temporal relationships with each other. They also strongly influenced other symptoms and were strongly influenced by other symptoms. In addition, *loneliness* influenced many other symptoms across time and especially itself. *Feeling depressed* was strongly influenced by other symptoms. *Not being happy* and *not enjoying life* had the lowest influence in the networks.

## Discussion

This is the first study to examine longitudinal symptom constellations of depression in older adults ages 50 and above by computing separate CLPN covering a timespan of 16 years (mean ages 65 to 76 years). Networks exhibited consistency over time. This is important as network results are not always perfectly replicable over time [17, 18, 30]. While networks in the present study were not completely identical, we discuss the most robust similarities across networks as the minor differences between networks appeared to be random and cannot be meaningfully attributed to specific developmental windows.

The symptom *feeling depressed* had strong incoming connections but fewer outgoing connections, consistent with prior network studies [28, 38]. We also found *sadness* had relatively few outgoing edges, as found in a longitudinal treatment study of adults completing weekly assessments [31]. Thus, other symptoms (e.g., *everything was an effort* or *loneliness*) are more likely to lead to *feeling depressed* or *sadness* than vice versa. Findings in adolescent and adult populations are however mixed, and some studies report more outgoing connections of *feeling depressed* in longitudinal network analyses [30, 55]. These studies differ from the present study in either the age range studied (most were younger), time-lags between assessments (most were shorter), and depressive symptoms assessed (different scales were used). While it is difficult to tease apart the predominant factor contributing to these cross-study differences, *feeling depressed* may have fewer outgoing connections as a function of increasing age. Specifically, *feeling depressed* may constitute a reaction to life changes (e.g., bereavement, decreased mobility, more health-related issues) that are less prevalent for younger people. Our findings that *feeling depressed* appears to be activated by other symptoms yet lacks outgoing connections is of clinical relevance, as depressed mood is one out of two hallmark symptoms required for diagnosis [10]. However, according to network analysis findings including the present study [28, 32, 56], when depressed mood is present, other symptoms and associated impairment are likely to have been experienced for some time. Thus, these precursor symptoms may be an important focus of early intervention efforts.

For instance, the symptoms *everything was an effort* and *could not get going* displayed many incoming and outgoing connections and were also strongly associated with each other and with *feeling depressed*. These symptoms were thus core symptoms of our networks, which underscores the relevance of somatic symptoms in the context of depression among older people in line with research on symptom presentations of older adults (>65 years of age) diagnosed with depressive episodes [57]. These symptoms could reflect fatigue or increased burden, which could be initiated by bereavement, pain, or decreased mobility, and may activate a further depressive symptom cascade. Such symptoms could stem from diverse sources such as lack of energy, lack of motivation, feeling sick, unable to concentrate, or the presence of other medical conditions [6]. They may also reflect higher levels of apathy that are more common with older age and prevalent in later-life depression [58]. This may reflect some disengagement from society and aligns with our finding that *everything was an effort* displayed strong bidirectional relationships with *loneliness*. Social support is important throughout the life course and may be crucial to master daily tasks when growing older [59]. Therefore, higher levels of *loneliness* in older people may precede the feeling that *everything was an effort*. In the other direction, individuals may reduce their social contacts if meeting people is exhausting. This could enhance feelings of *loneliness* and thus *everything was an effort* and *loneliness* could be mutually reinforcing.

Overall, *loneliness* was an important symptom with many incoming and outgoing connections, and strongly predicted itself over time. This supports the observation that older people are often socially isolated [6]. As discussed above, loneliness can be increased by physical symptoms unrelated to depression in older adults [60]. The outgoing connections towards *feeling depressed* or *sadness* accord with previous research that loneliness in older adults predicted total scores of depressive symptoms one year later [61]. To extend our knowledge of loneliness in older adulthood, this symptom should be investigated in the context of separation, bereavement, and widowhood [62]. In addition, future work should scrutinize whether this symptom’s centrality differs as a function of feeling lonely versus being isolated due to one’s circumstances. In the broader literature, loneliness emerged as core symptom in adolescent depression networks [63–66] and was strongly associated with depressed affect in adults [67]. This suggests that loneliness is a core experience that leads to increased vulnerability to depression across the life course [6, 66, 68–70]. Sensitivity to social exclusion and the need for social connection appear to be fundamentally linked to depression across different developmental periods [34, 69, 71].

At most waves, *restless sleep* displayed outgoing connections but fewer incoming associations, in line with the only prior longitudinal network study in older adults where trouble sleeping had some outgoing connections but almost no incoming associations [28]. This item also strongly predicted itself over time. Results regarding this symptom were mixed in previous cross-sectional samples of older adults which could not disentangle incoming and outgoing connections [26]. As *restless sleep* can capture a variety of sleep difficulties (e.g., insomnia, parasomnia, restless leg syndrome), this symptom needs clarification in future studies. Within our network perspective, the overarching term *restless sleep* seemed to contribute to *everything was an effort* and *could not get going*, which, in turn, contributed to *feeling depressed*. This result aligns with a somatic pathway to depressive symptoms in older populations [8], and the association of disrupted sleep with the course of depression and treatment outcomes [36]. *Restless sleep* could be a potential warning symptom of depression in older adults, useful in primary prevention interventions [6]. However, this may not be specific to depression as restless sleep is considered a precursor to many forms of psychopathology [35].

*Not enjoying life* and *not being happy* [6] were strongly correlated with each other but were the least central symptoms on both centrality measures, which could be a byproduct of the reverse coding of these items. Around 90% of participants endorsed these items, resulting in the restricted range of variance, which can influence network centrality. It is probable that a substantial proportion of the variance in *enjoying life* is accounted for by *happiness*, and vice versa. This would lead to limited unique variance in either item, thereby resulting in minimal associations between *enjoying life* and other symptoms when controlling for *happiness*, and vice versa. Furthermore, the high degree of conceptual overlap between the two items may also result in topological overlap [29].

### Clinical implications

While network studies can inform of central symptoms to target for interventions, significant associations between symptoms may not be clinically meaningful for several reasons [32, 72]. First, as associations have been found in general populations, network studies must be conducted in clinical populations to discern whether such associations hold. Second, even in clinical populations it remains unknown whether intervening on these central elements would be associated with symptom improvement let alone alleviating functional impairment. In theory, targeting core symptoms in a network should reduce overall network connectivity, but this has yet to be examined. Third, there is no consensus on what effect sizes between symptoms in a network are considered clinically meaningful. While smaller effect sizes may be meaningful on a population level, larger effect sizes are likely necessary in clinical populations to counteract functional impairment associated with depressive symptoms [73].

Nonetheless, central symptoms that consistently emerged in our networks over time (*everything was an effort, could not get going, loneliness*) may be potential key targets to mitigate depression in older adults on a population level [72]. These findings are of particular importance given their consistency across multiple time periods, which suggests the existence of shared processes that may be targeted at various developmental stages. Importantly, none of these symptoms were measured in the only prior longitudinal depression network study on older adults which we are aware of [28]. As these symptoms are not specific to depression but occur in the context of multiple other mental and physical disorders, they may even constitute viable transdiagnostic targets. *Everything was an effort* and *could not get going* may be targeted through behavioral activation (e.g., encouraging people to engage in activities). Setting achievable goals and engaging in meaningful activities that align with an individual’s values and interests can help individuals overcome the sense of everything being an effort and increase their enjoyment in daily life. To prevent and counteract the effects of loneliness, interventions have been proposed at the individual level (e.g., increasing social skills or increasing opportunities for social interaction) and societal level (e.g., targeting structures in educational and institutional settings) [74]. Our research should therefore stimulate intervention studies that empirically test whether targeting these symptoms leads to overall symptom reduction.

### Strengths & limitations

Our longitudinal network analyses contributes significantly to the literature by disentangling incoming and outgoing connections of depressive symptoms over nine waves in a representative sample of older adults.

Limitations are as follows. First, findings from CLPN analysis may be biased since stable individual differences (between-person effects) are not disaggregated from within-person effects [47]. Thus, our results solely indicate that individuals who have higher levels of core symptoms (e.g., could not get going) are more likely to endorse other symptoms at the next time point. However, this does not reveal whether individuals with higher core symptoms than usual will experience a subsequent increase in other symptoms. Disaggregation of within- and between-person variances is possible with multilevel vector autoregression networks models in the context of experience sampling studies [54]. However, for such analyses to adequately capture within-person associations, many more waves of data are required than available in the present study. Furthermore, interpretation of these more complex models can be challenging [61], and in the absence of a control group, conclusions that one symptom causes another symptom to change are unfounded. Second, the present two-year time frame between assessments may occlude some shorter-term associations between symptoms. The level of symptom fluctuation within these two years is unknown and at the point of assessment we cannot discern whether the networks reflect cumulative change over two years or random fluctuations. However, the consistency of the results across networks over time points to enduring patterns of symptom prediction. Nonetheless, studies with shorter time-intervals, for example in the context of typical diagnostic measures (i.e., two weeks), or even on a daily basis, are desirable to capture finer-grained associations over time. Third, CES-D-8 only measures eight depressive symptoms, which are not exhaustive of this condition [75]. Core concepts like loneliness, somatic symptoms, or sleep problems were not captured in their entire breath which may have obfuscated more nuanced insights into the interrelatedness of these symptoms. Fourth, items were assessed with a dichotomous response format, potentially restricting their variance, which may have led to weaker associations among items than a continuous response format. Fifth, attrition was substantial in our sample. While we included variables associated with missingness in our imputation model, unmeasured variables may have contributed to attrition. Our imputed data are only unbiased if our data are missing at random [46]. Finally, the present contribution is limited to the ethnically white majority population of England [76]. This limitation is further compounded by the fact that ethnic minority status predicted study dropout.

## Conclusion

The present study illuminates consistent longitudinal relationships between depressive symptoms in people 50 years and older. *Everything was an effort, could not get going* and *loneliness* emerged as key symptoms, which may serve as a starting point for further network analyses or intervention studies.

### Supplementary information

Supplemental Material 1

Supplemental Material 2

Supplemental Material 3

## Data Availability

Data are openly available to researchers via the UK Data Service.
