# Supplementary material for: The development of depressive symptoms in older adults from a network perspective in the English Longitudinal Study of Ageing
Source: Transl Psychiatry. 2023 Nov 25;13:363. doi: 10.1038/s41398-023-02659-0 (PMC10676393; doi:10.1038/s41398-023-02659-0)
Supplement: Supplementary file 1 — Supplemental Material 1 [file 41398_2023_2659_MOESM1_ESM.docx]

| 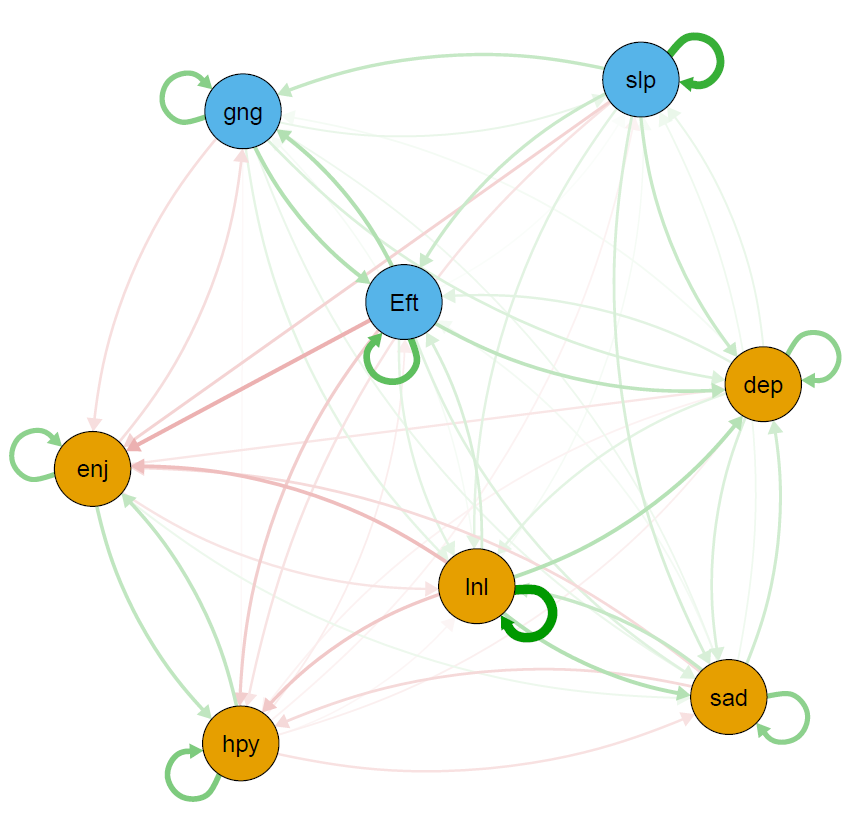  T1 → T2 | 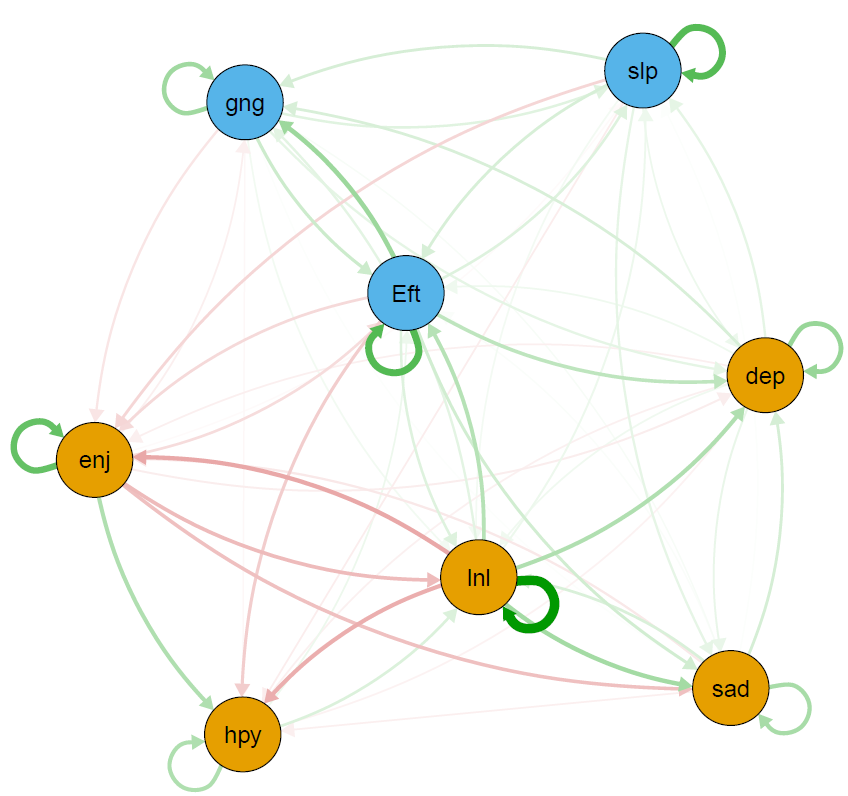  T2 → T3 | 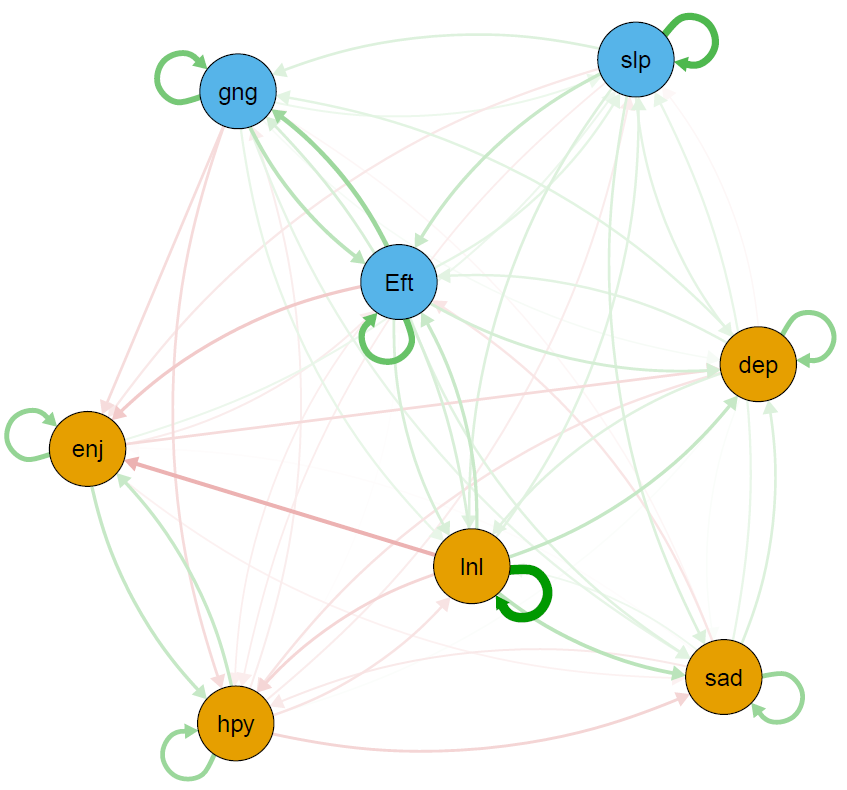  T3 → T4 | 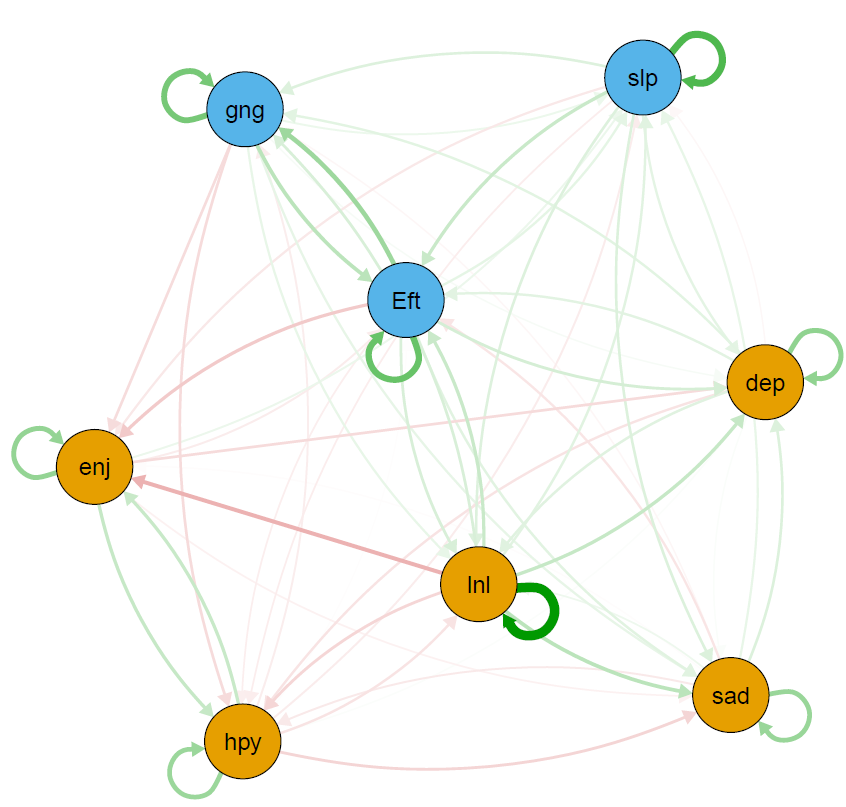  T4 → T5 |
| --- | --- | --- | --- |
| 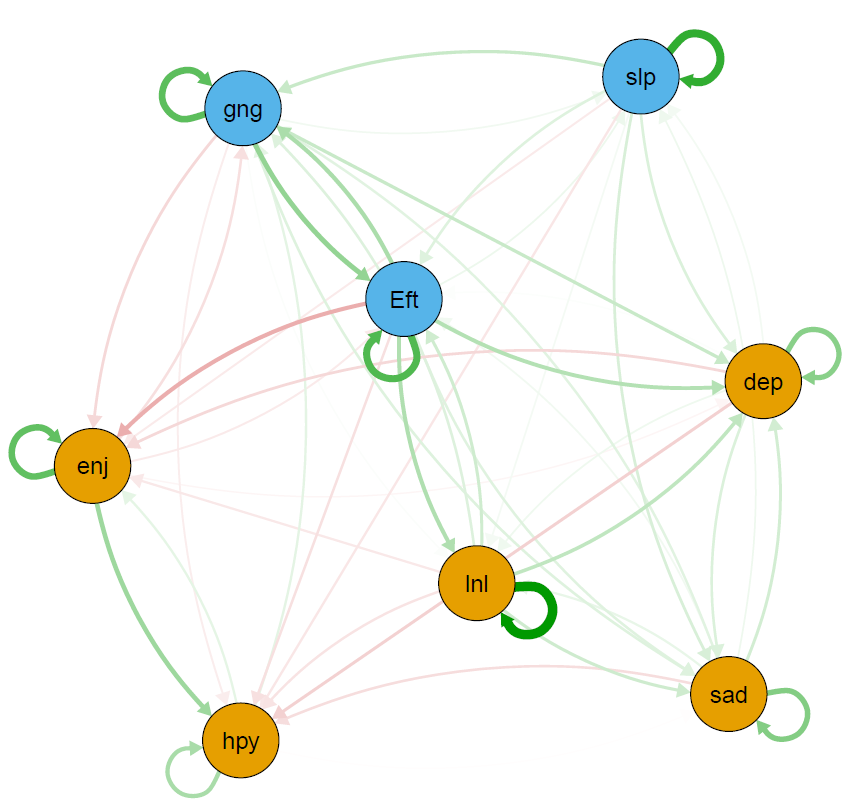  T5 → T6 | 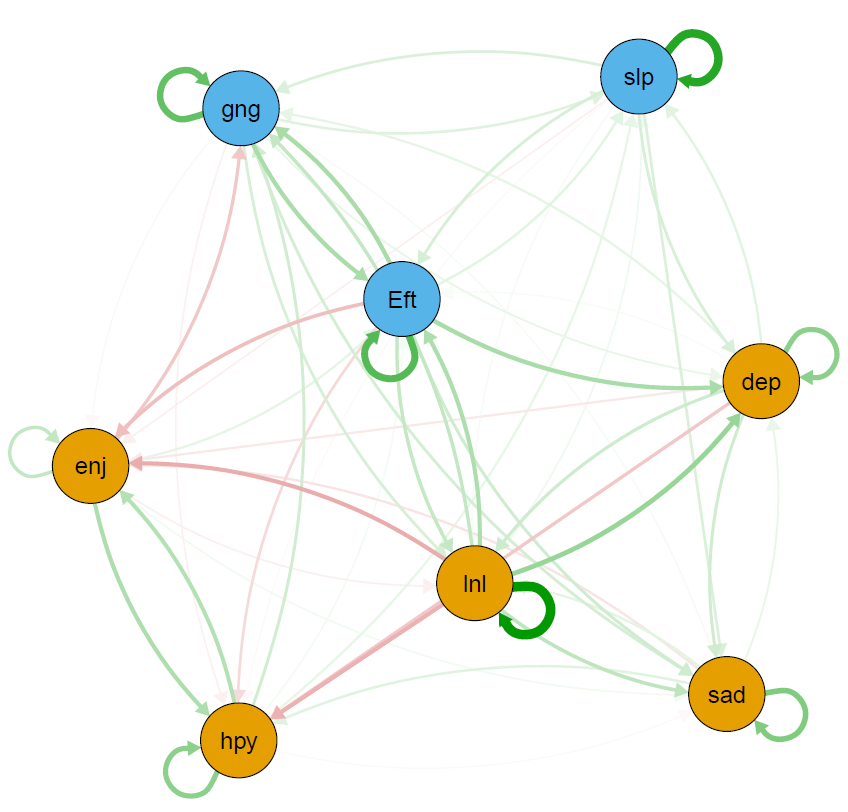  T6 → T7 | 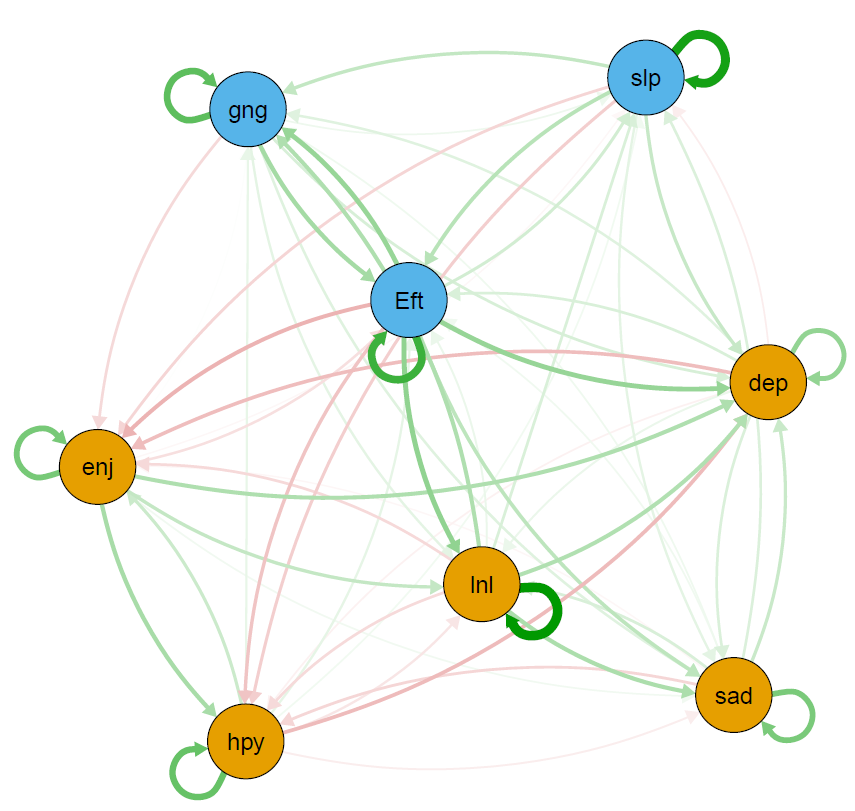  T7 → T8 | 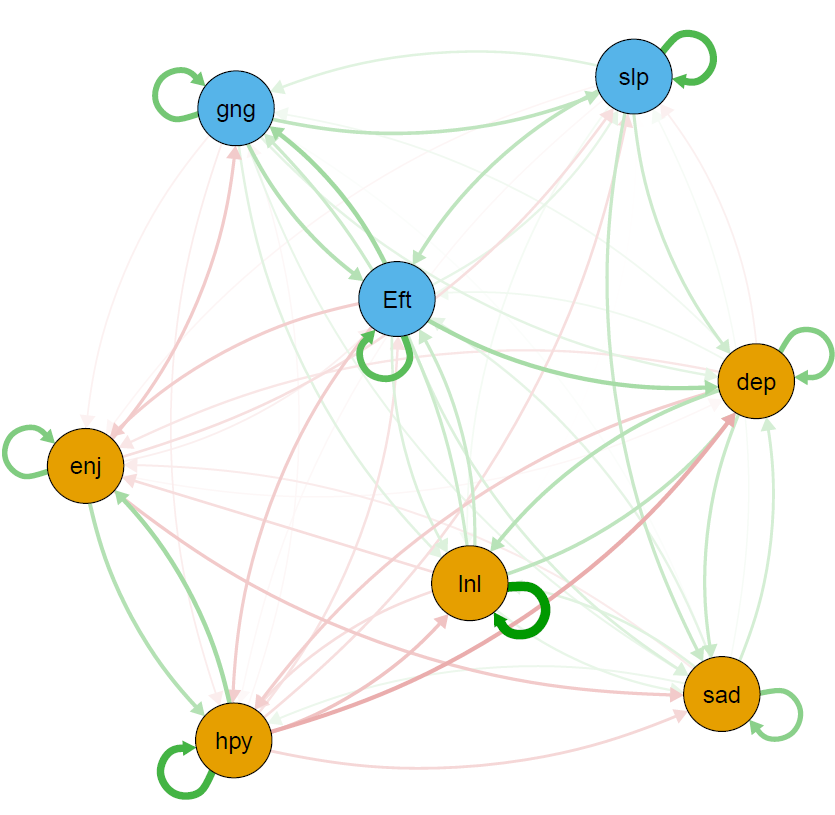  T8 → T9 |

**Figure s1.** The cross-lagged panel networks for consecutive time-points displaying autoregressive effects.

Dep = felt depressed, Eft = everything you did was an effort, slp = restless sleep, hyp = happy, lnl = lonely, enj = enjoyed life, sad = felt sad, gng = could not get going. Arrows represent unique longitudinal relationships. Green edges indicate positive relationships; red edges indicate negative relationships (note that there are negative relationships as happy and enjoyed life were coded in the opposite direction as the other items). Edge thickness displays the relationship strength. Autoregressive edges and covariates were excluded to enhance visual interpretation. Yellow nodes represent symptoms of “depressed affect”. Blue nodes represent symptoms of “somatic complaints”. Non-significant cross-lagged paths are excluded.

All networks were visualized with an average layout using the qgraph package (Epskamp et al., 2012). Nodes represent symptoms and arrows represent estimates of cross-lagged effects. The color of the arrows represents the directionality of the effect (green = positive effect, red = negative effect). Thicker arrows indicate stronger effects; non-significant cross-lagged paths were excluded. Nodes that cluster more strongly are placed together in the graph (Fruchterman & Reingold, 1991). For better visual interpretation, nodes were colored according to the two-factor solution of the CES-D scale (depressed affect & somatic complaints). The underlying algorithm visualizes line thickness as a function of the strongest paths.

| 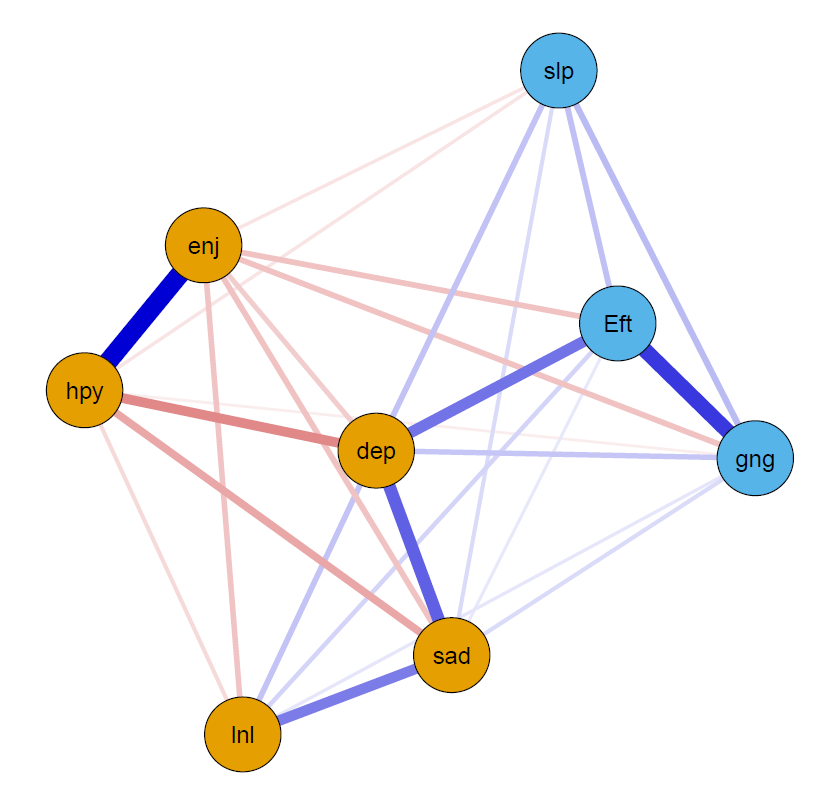  Wave 1 | 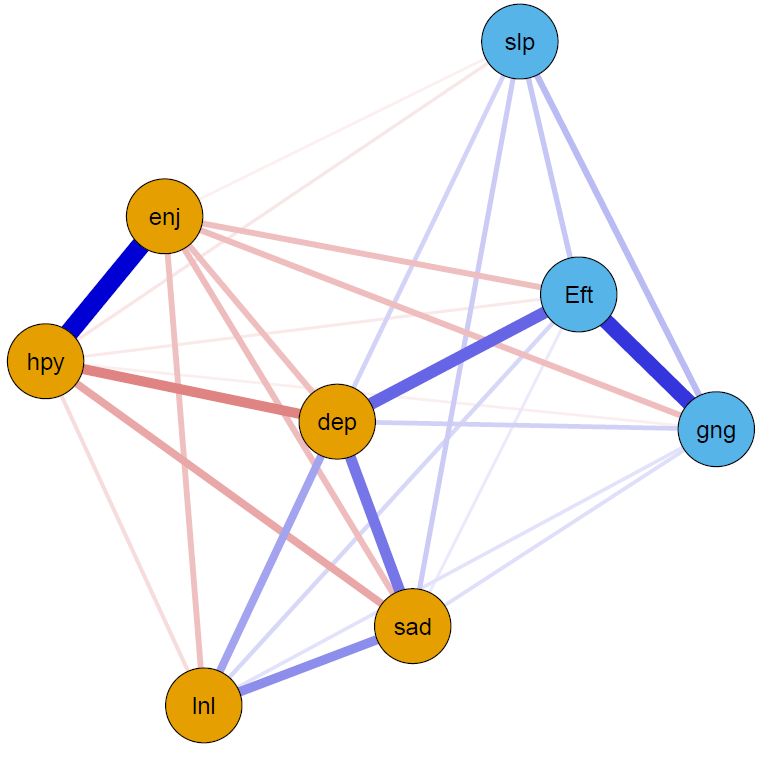  Wave 2 | 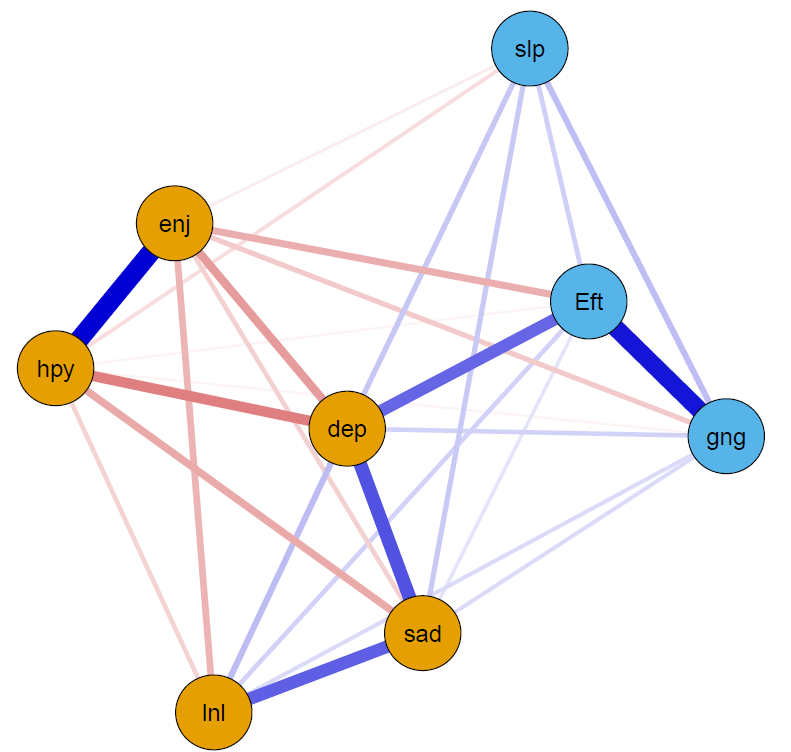  Wave 3 |
| --- | --- | --- |
| 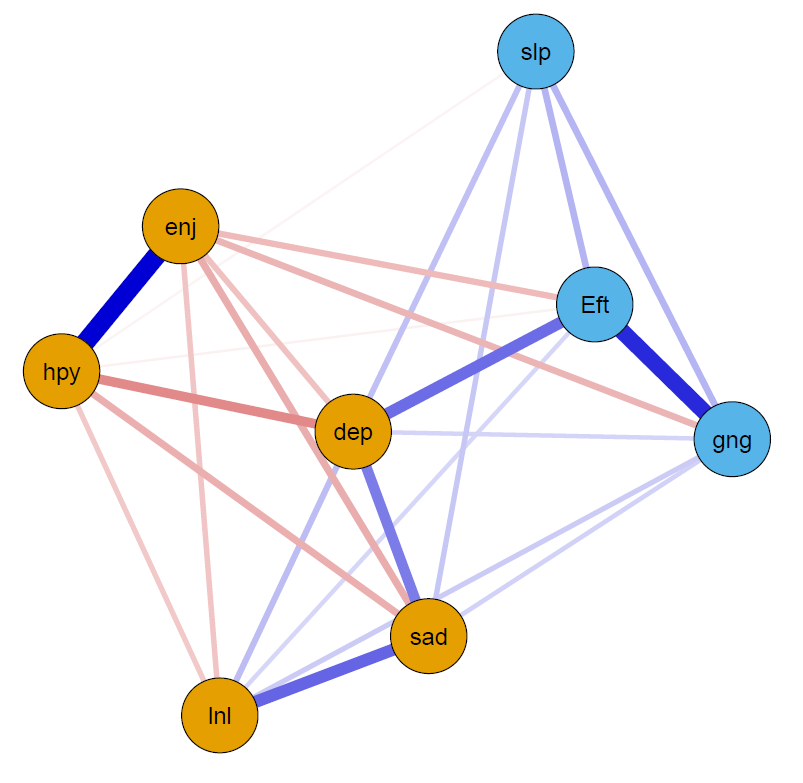  Wave 4 | 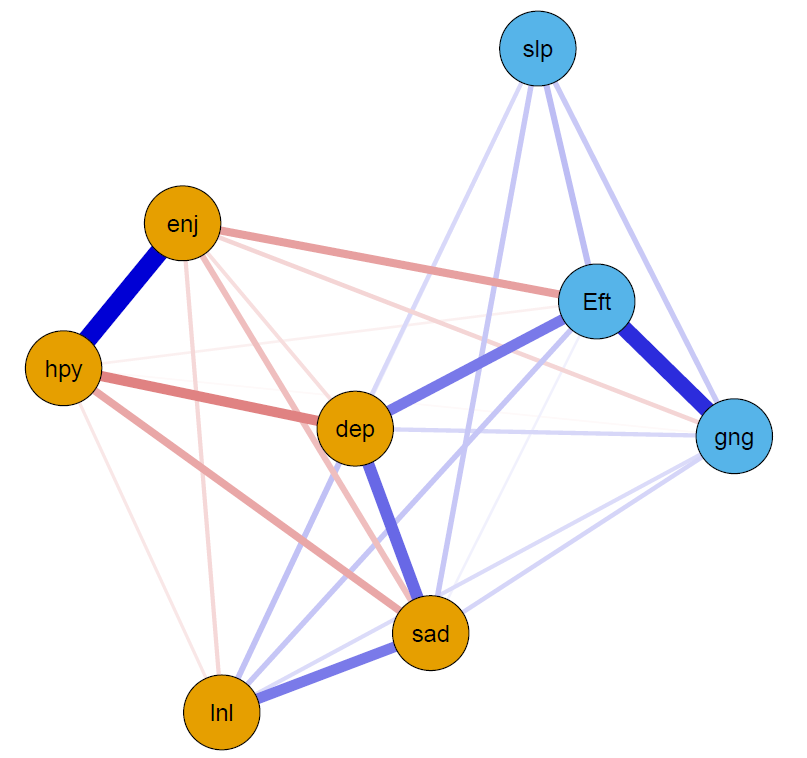  Wave 5 | 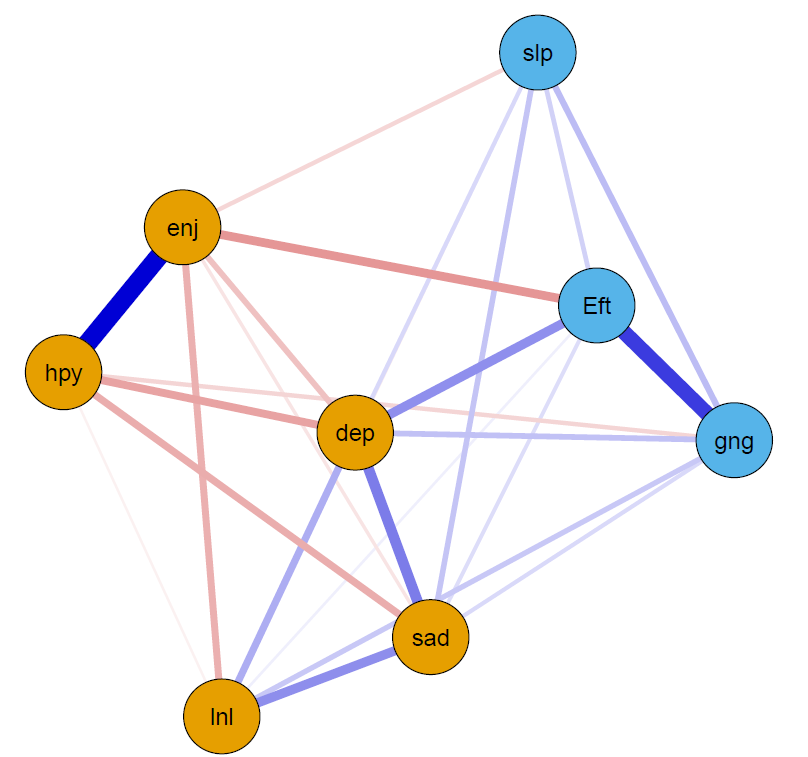  Wave 6 |
| 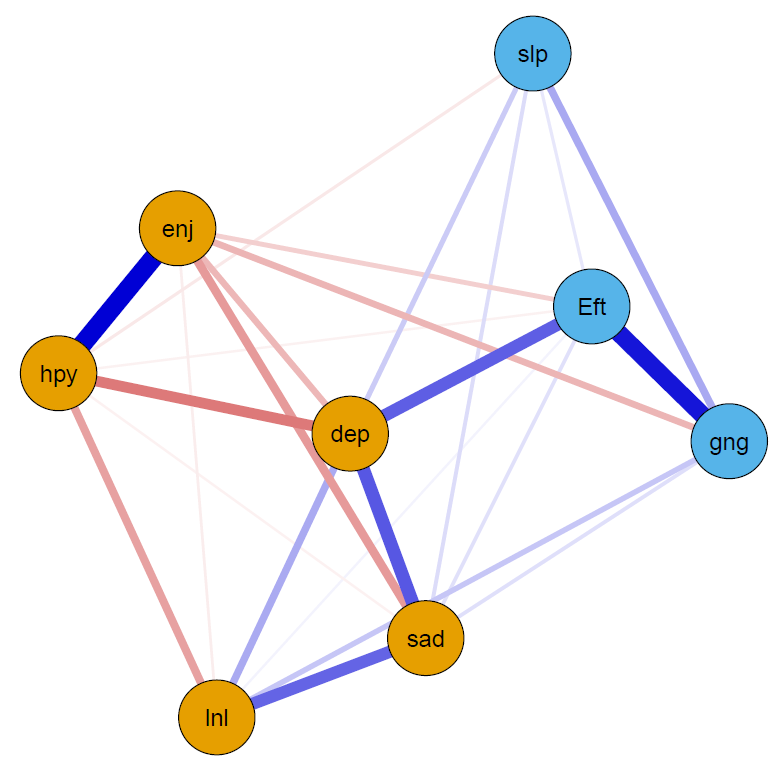  Wave 7 | 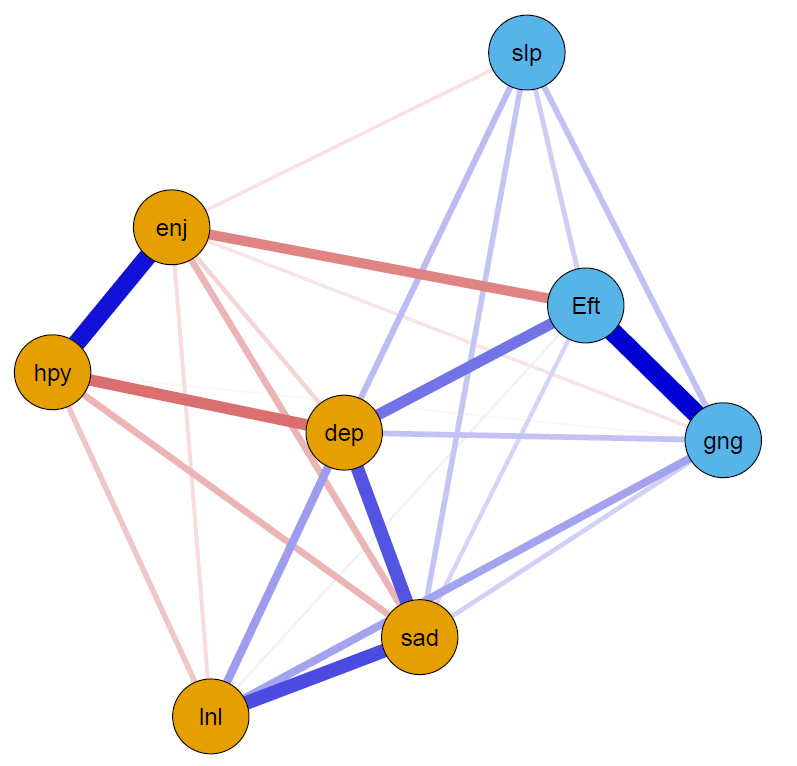  Wave 8 | 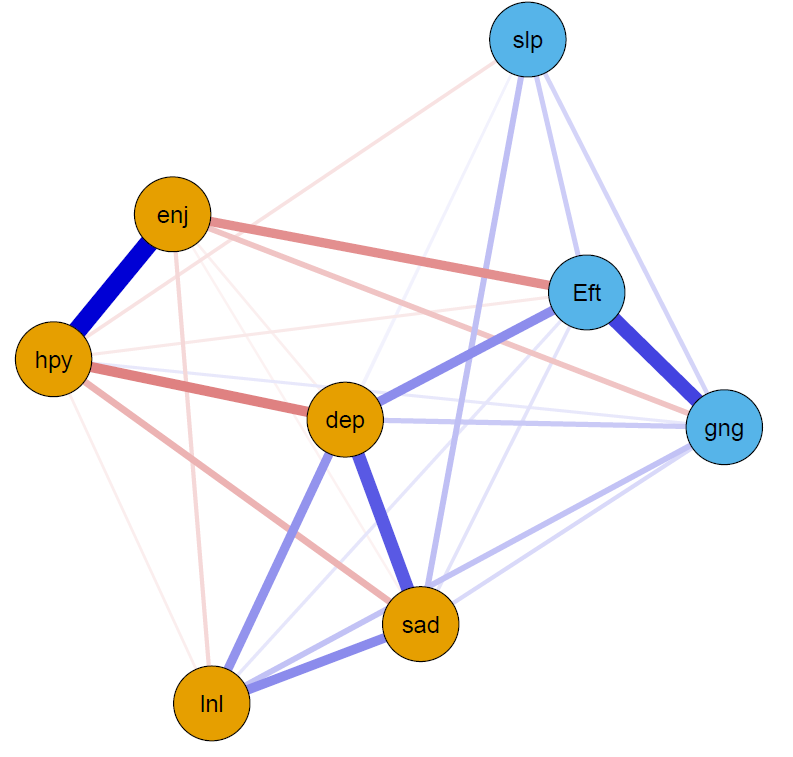  Wave 9 |

**Figure s2.** Cross sectional networks for all waves (Ising Models)

Dep = felt depressed, Eft = everything you did was an effort, slp = restless sleep, hyp = happy, lnl = lonely, enj = enjoyed life, sad = felt sad, gng = could not get going. Arrows represent unique longitudinal relationships. Blue edges indicate positive relationships; red edges indicate negative relationships (note that there are negative relationships as happy and enjoyed life were coded in the opposite direction as the other items). Edge thickness displays the relationship strength. Yellow nodes represent symptoms of “depressed affect”. Blue nodes represent symptoms of “somatic complaints”. Non-significant cross-lagged paths are excluded.

All networks were visualized with an average layout using the qgraph package using the ising fit method (Epskamp et al., 2012). Nodes represent symptoms and arrows represent estimates of partial correlation. The color of the arrows represents the directionality of the effect (green = positive effect, red = negative effect). Thicker arrows indicate stronger effects; non-significant cross-lagged paths were excluded. Nodes that cluster more strongly are placed together in the graph (Fruchterman & Reingold, 1991). For better visual interpretation, nodes were colored according to the two-factor solution of the CES-D scale (depressed affect & somatic complaints). The underlying algorithm visualizes line thickness as a function of the strongest paths.


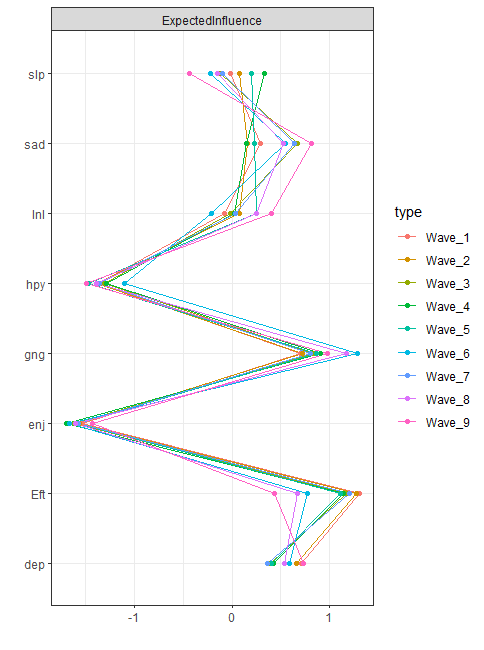


**Figure s3.** Symptom centrality (Expected Influence) estimates for the networks using z-values. Greater values indicate greater centrality. Dep = felt depressed, Eft = everything you did was an effort, slp = restless sleep, hyp = happy, lnl = lonely, enj = enjoyed life, sad = felt sad, gng = could not get going. Type refers to waves used in each network model (t = timepoint).


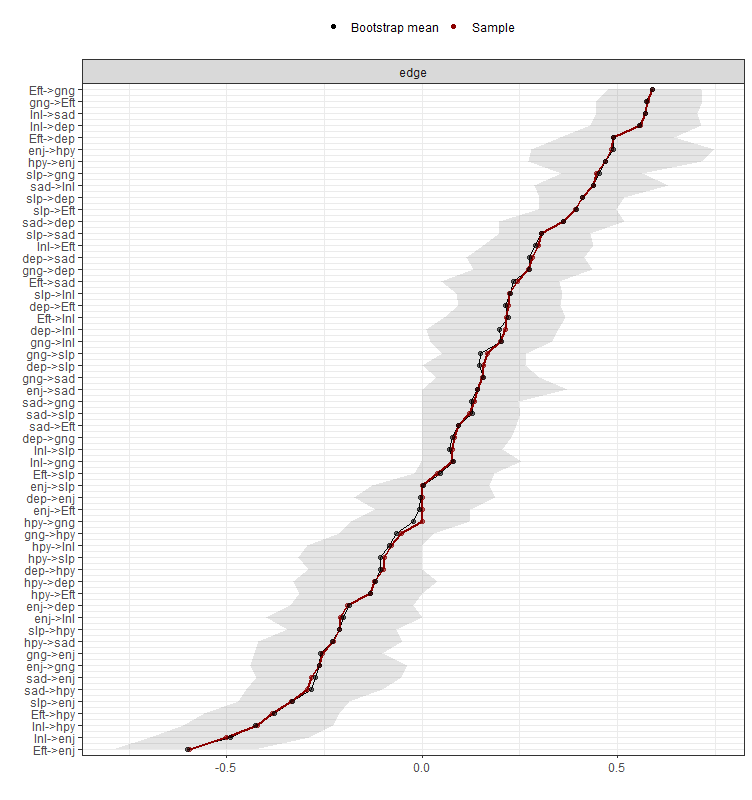


**Figure s4.** CES-D interrelation accuracy plots with 1000 bootstrap iterations for the T1 → T2 network. Plots show the sample interrelations (i.e., edge weights) with the red dots, the means of the bootstrapped interrelations (i.e., edge weights) with black dots, and the bootstrap confidence intervals.

Dep = felt depressed, Eft = everything you did was an effort, slp = restless sleep, hyp = happy, lnl = lonely, enj = enjoyed life, sad = felt sad, gng = could not get going.


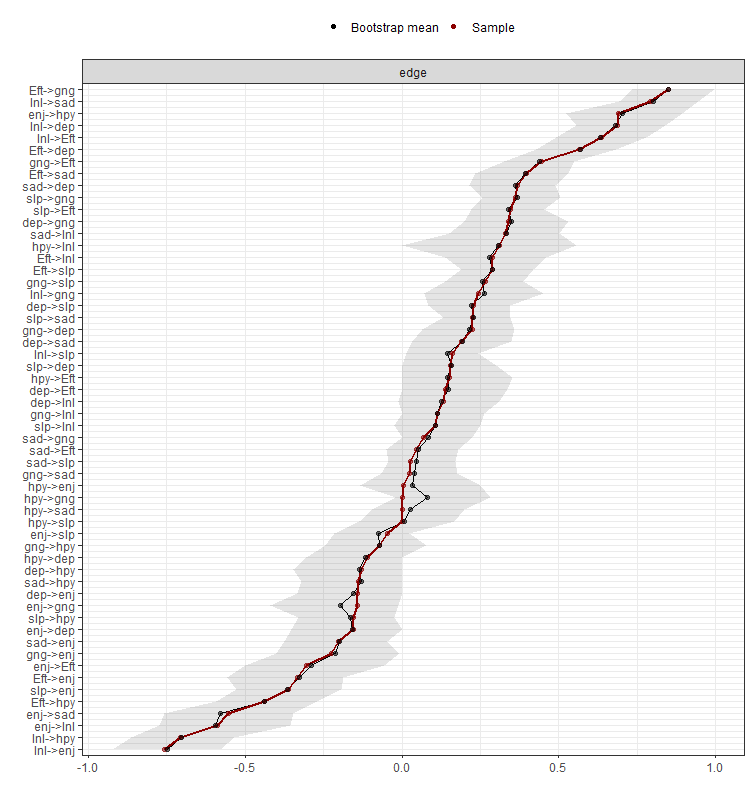


**Figure s5.** CES-D interrelation accuracy plots with 1000 bootstrap iterations for the T2 → T3 network. Plots show the sample interrelations (i.e., edge weights) with the red dots, the means of the bootstrapped interrelations (i.e., edge weights) with black dots, and the bootstrap confidence intervals.

Dep = felt depressed, Eft = everything you did was an effort, slp = restless sleep, hyp = happy, lnl = lonely, enj = enjoyed life, sad = felt sad, gng = could not get going.


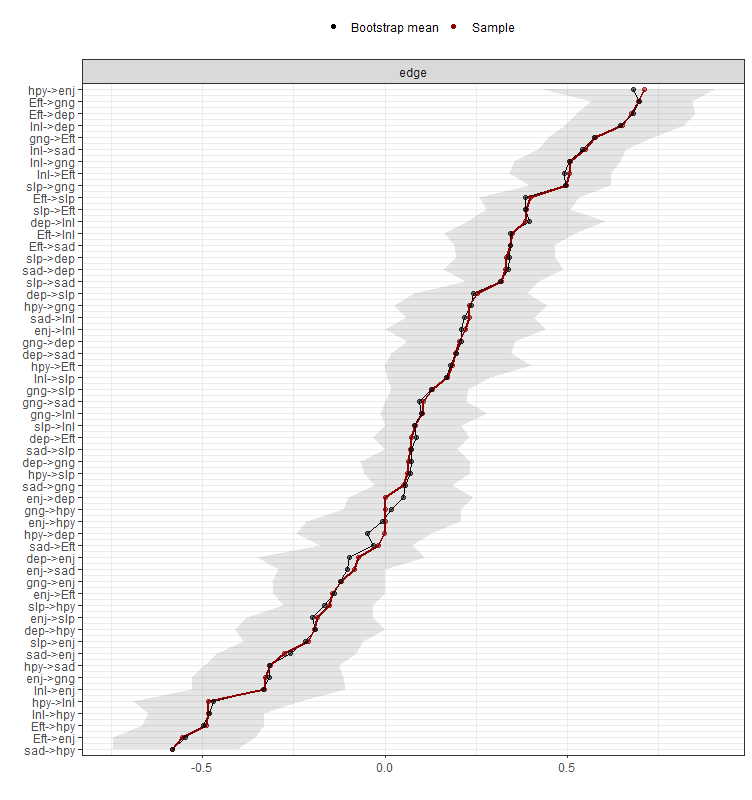


**Figure s6.** CES-D interrelation accuracy plots with 1000 bootstrap iterations for the T3 → T4 network. Plots show the sample interrelations (i.e., edge weights) with the red dots, the means of the bootstrapped interrelations (i.e., edge weights) with black dots, and the bootstrap confidence intervals.

Dep = felt depressed, Eft = everything you did was an effort, slp = restless sleep, hyp = happy, lnl = lonely, enj = enjoyed life, sad = felt sad, gng = could not get going.


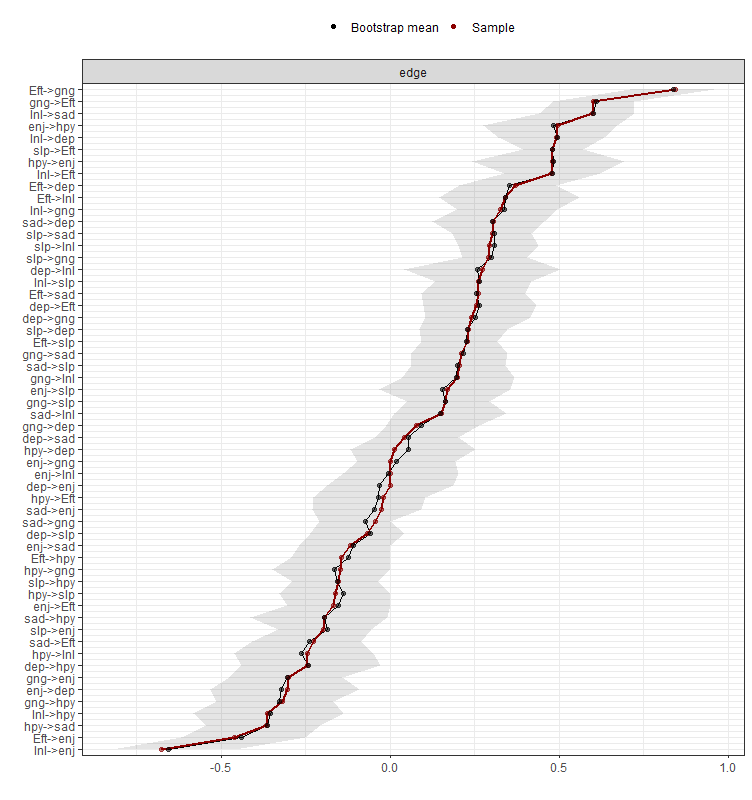


**Figure s7.** CES-D interrelation accuracy plots with 1000 bootstrap iterations for the T4 → T5 network. Plots show the sample interrelations (i.e., edge weights) with the red dots, the means of the bootstrapped interrelations (i.e., edge weights) with black dots, and the bootstrap confidence intervals.

Dep = felt depressed, Eft = everything you did was an effort, slp = restless sleep, hyp = happy, lnl = lonely, enj = enjoyed life, sad = felt sad, gng = could not get going.


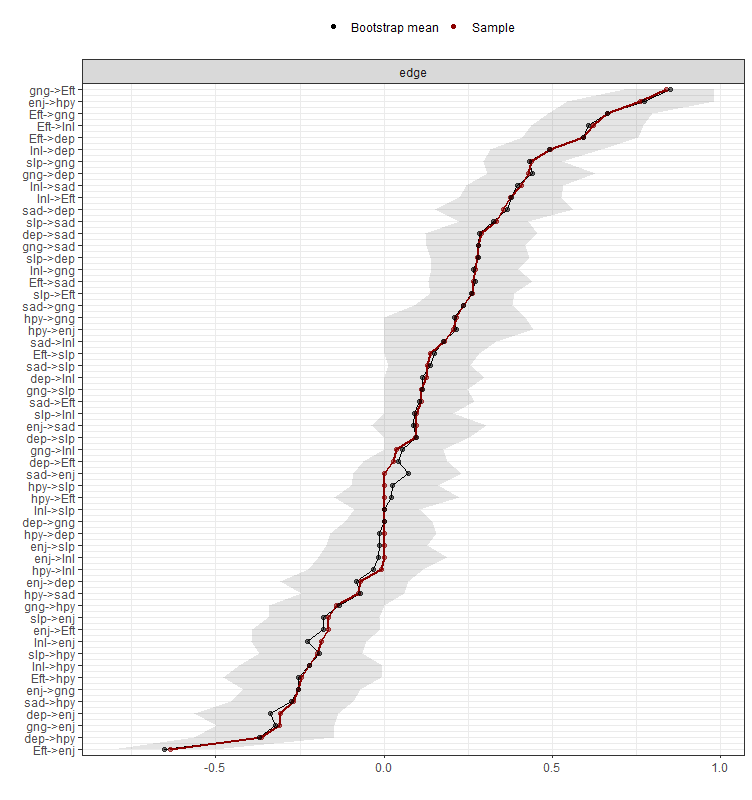


**Figure s8.** CES-D interrelation accuracy plots with 1000 bootstrap iterations for the T5 → T6 network. Plots show the sample interrelations (i.e., edge weights) with the red dots, the means of the bootstrapped interrelations (i.e., edge weights) with black dots, and the bootstrap confidence intervals.

Dep = felt depressed, Eft = everything you did was an effort, slp = restless sleep, hyp = happy, lnl = lonely, enj = enjoyed life, sad = felt sad, gng = could not get going.


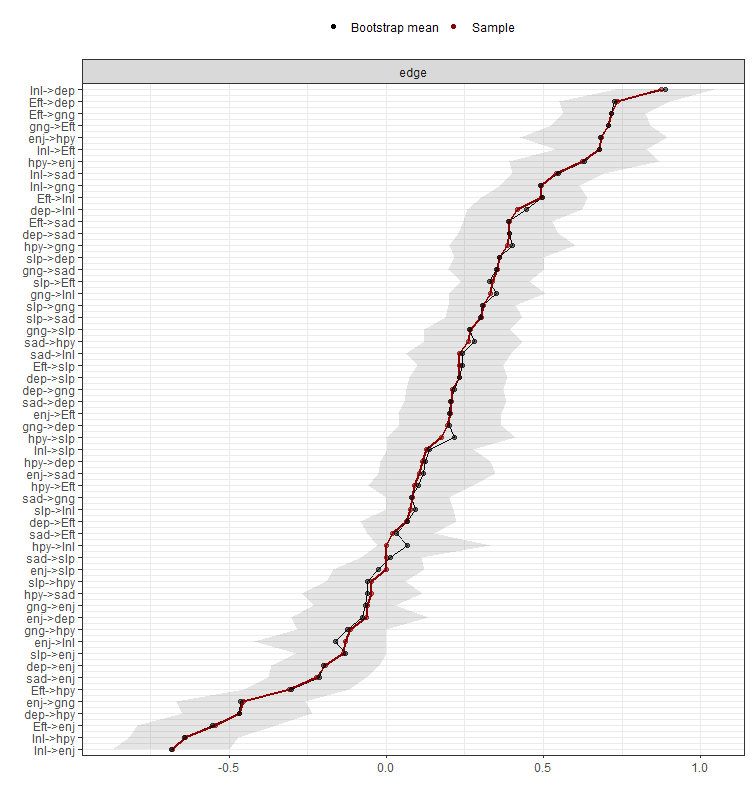


**Figure s9.** CES-D interrelation accuracy plots with 1000 bootstrap iterations for the T6 → T7 network. Plots show the sample interrelations (i.e., edge weights) with the red dots, the means of the bootstrapped interrelations (i.e., edge weights) with black dots, and the bootstrap confidence intervals.

Dep = felt depressed, Eft = everything you did was an effort, slp = restless sleep, hyp = happy, lnl = lonely, enj = enjoyed life, sad = felt sad, gng = could not get going.


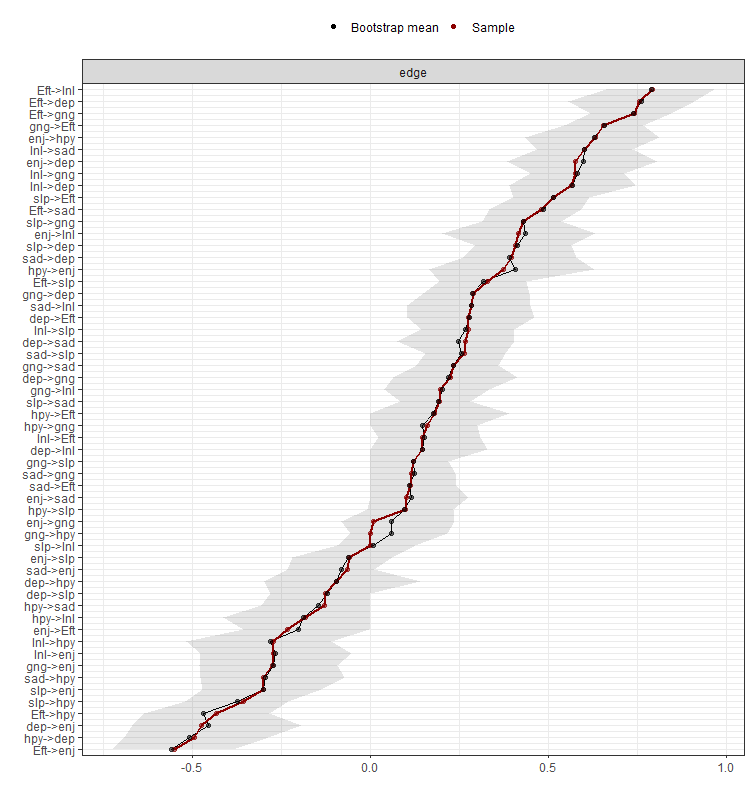


**Figure s10.** CES-D interrelation accuracy plots with 1000 bootstrap iterations for the T7 → T8 network. Plots show the sample interrelations (i.e., edge weights) with the red dots, the means of the bootstrapped interrelations (i.e., edge weights) with black dots, and the bootstrap confidence intervals.

Dep = felt depressed, Eft = everything you did was an effort, slp = restless sleep, hyp = happy, lnl = lonely, enj = enjoyed life, sad = felt sad, gng = could not get going.


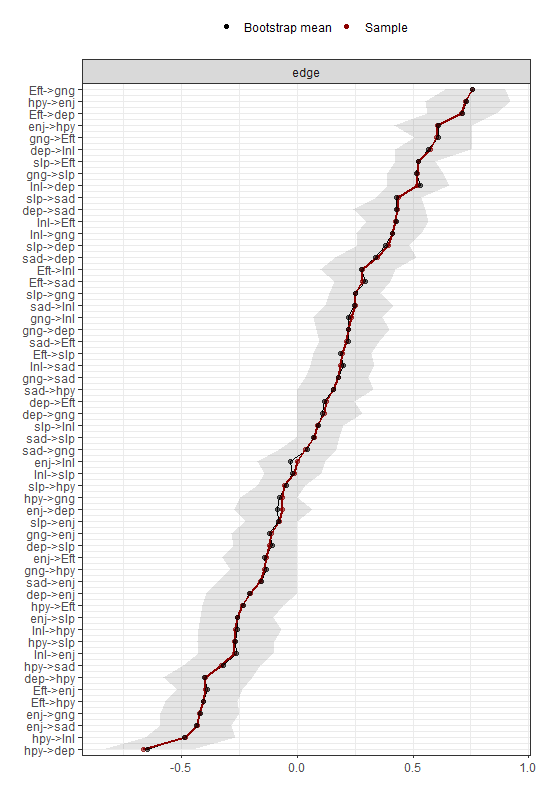


**Figure s11.** CES-D interrelation accuracy plots with 1000 bootstrap iterations for the T8 → T9 network. Plots show the sample interrelations (i.e., edge weights) with the red dots, the means of the bootstrapped interrelations (i.e., edge weights) with black dots, and the bootstrap confidence intervals.

Dep = felt depressed, Eft = everything you did was an effort, slp = restless sleep, hyp = happy, lnl = lonely, enj = enjoyed life, sad = felt sad, gng = could not get going.


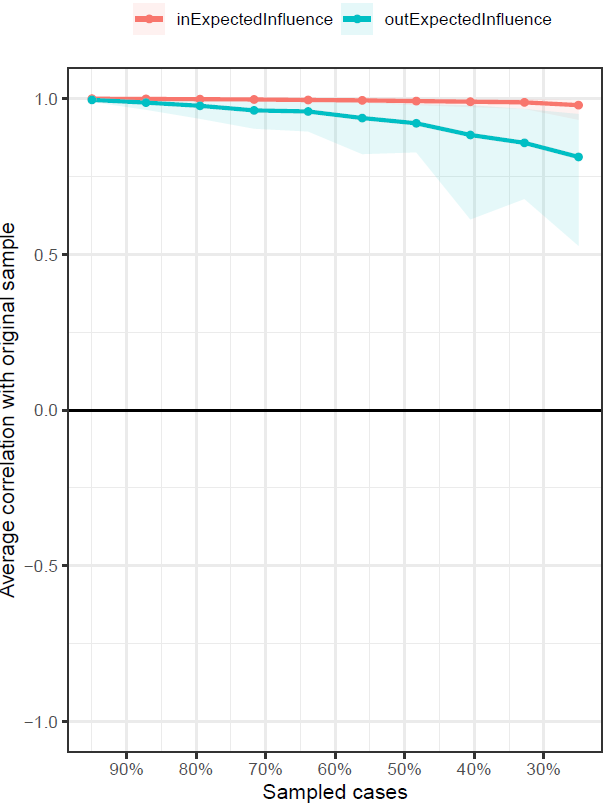


**Figure s12.** Stability of centrality measures for the T1 → T2 network


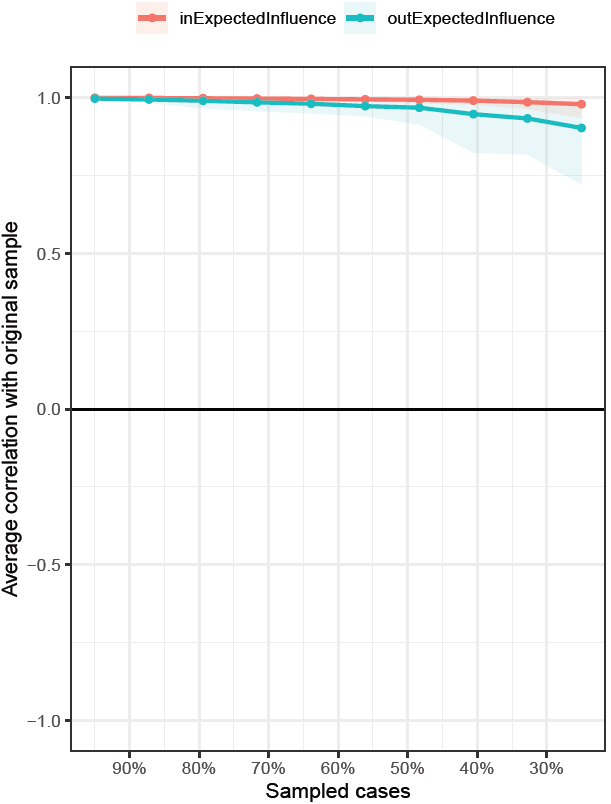


**Figure s13.** Stability of centrality measures for the T2 → T3 network


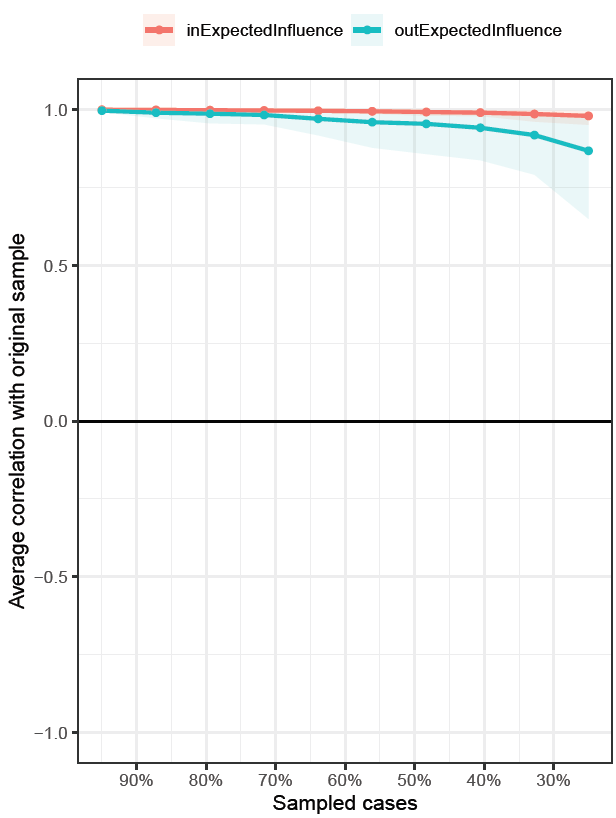


**Figure s14.** Stability of centrality measures for the T3 → T4 network


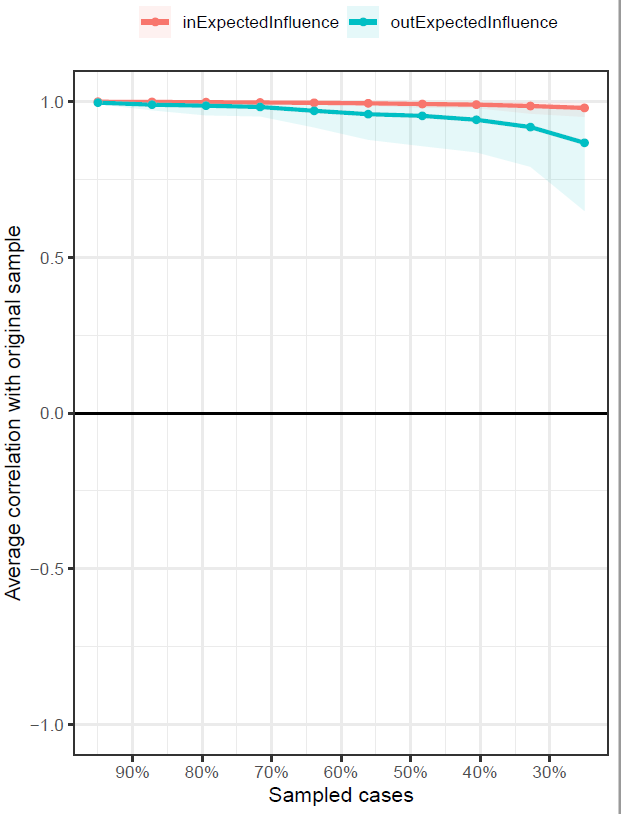


**Figure s15.** Stability of centrality measures for the T4 → T5 network


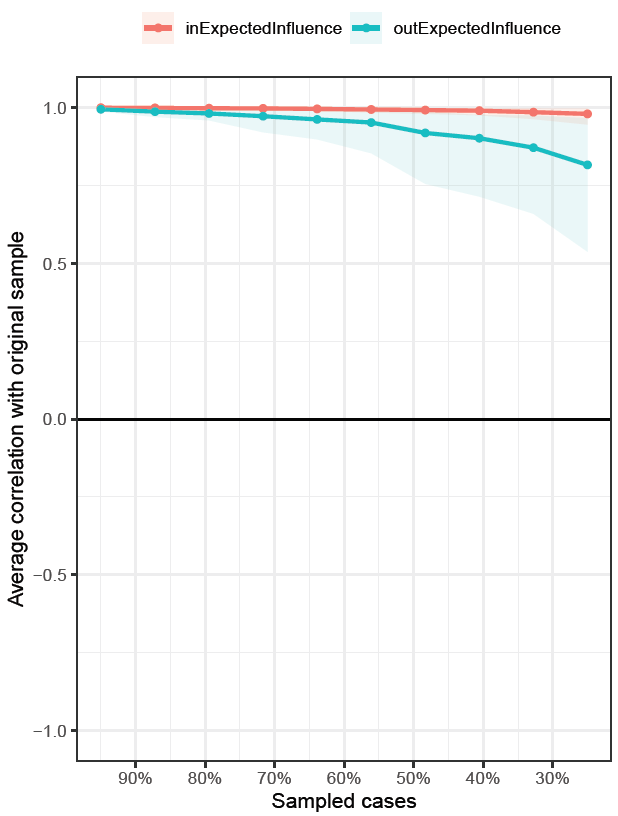


**Figure s16.** Stability of centrality measures for the T5 → T6 network


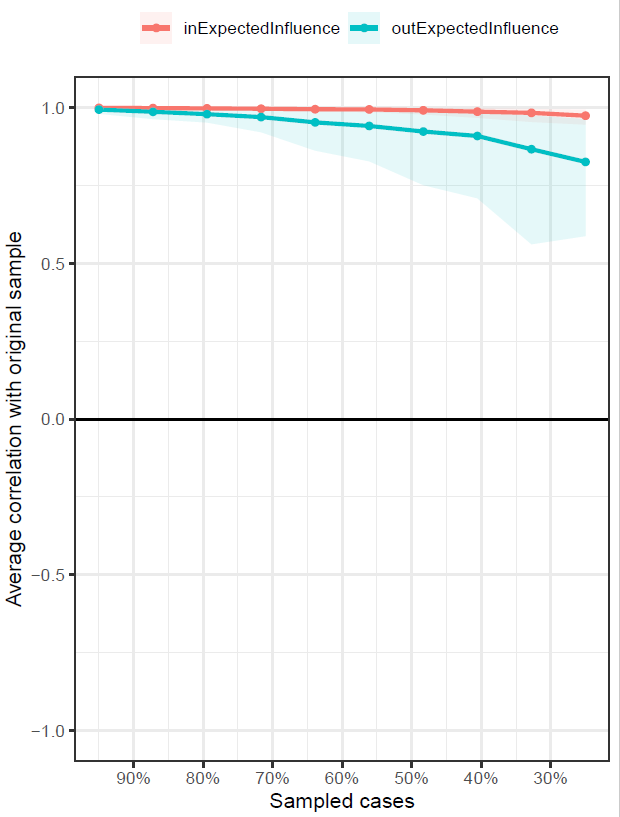


**Figure s17.** Stability of centrality measures for the T6 → T7 network


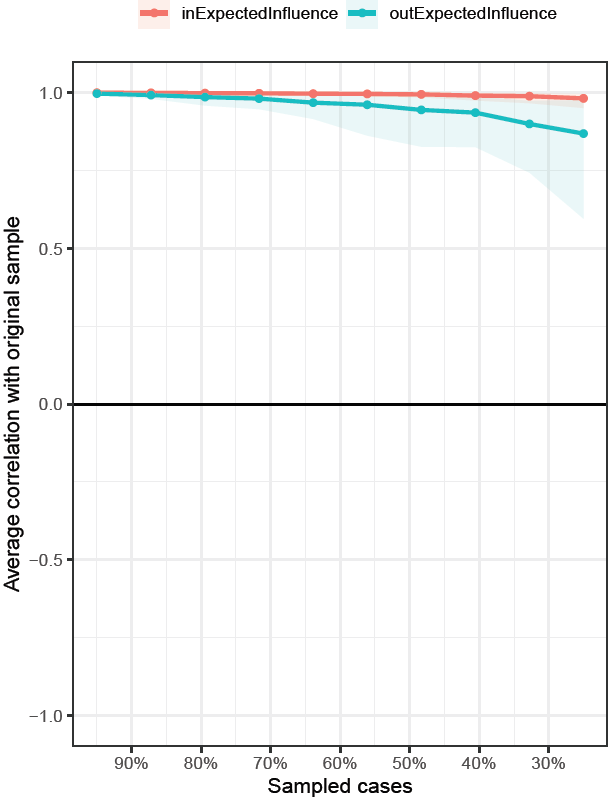


**Figure s18.** Stability of centrality measures for the T7 → T8 network


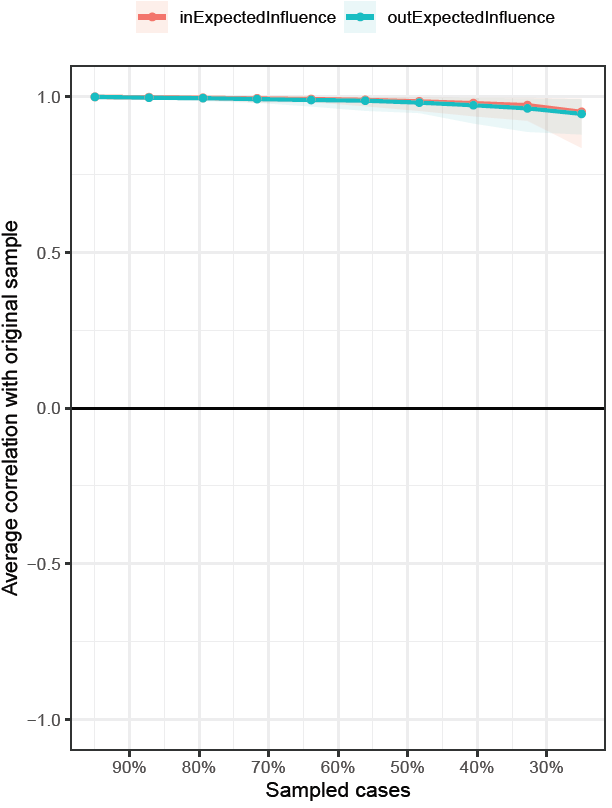


**Figure s19.** Stability of centrality measures for the T8 → T9 network


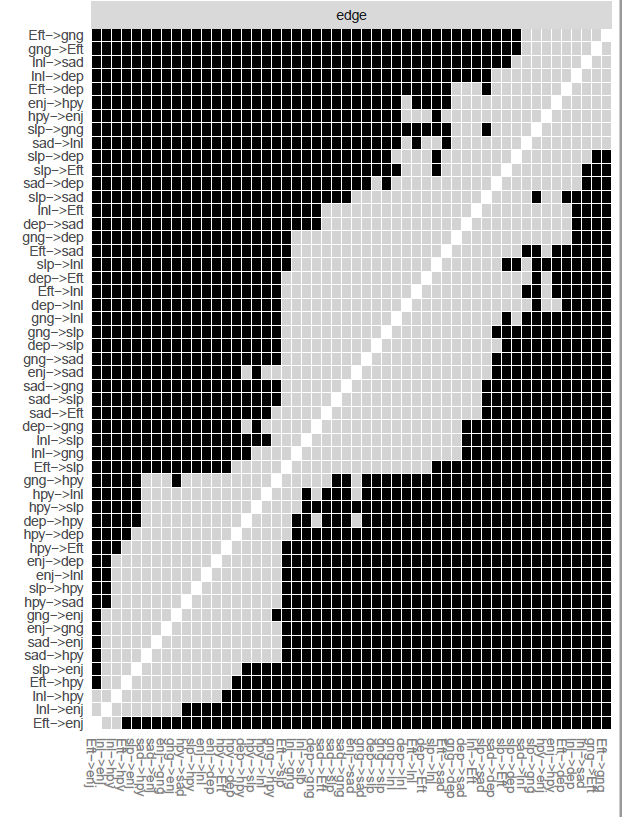


**Figure s20.** Edge weight difference tests for the T1 → T2 network. Black boxes indicate edges that significantly differ from each other (*p* < .05). Gray boxes indicate no differences.

Dep = felt depressed, Eft = everything you did was an effort, slp = restless sleep, hyp = happy, lnl = lonely, enj = enjoyed life, sad = felt sad, gng = could not get going.


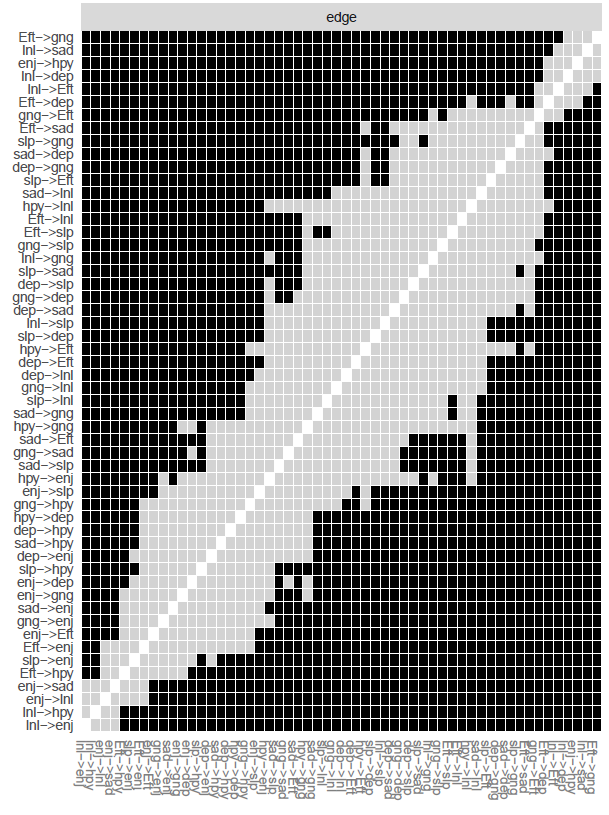


**Figure s21.** Edge weight difference tests for the T2 → T3 network. Black boxes indicate edges that significantly differ from each other (*p* < .05). Gray boxes indicate no differences.

Dep = felt depressed, Eft = everything you did was an effort, slp = restless sleep, hyp = happy, lnl = lonely, enj = enjoyed life, sad = felt sad, gng = could not get going.


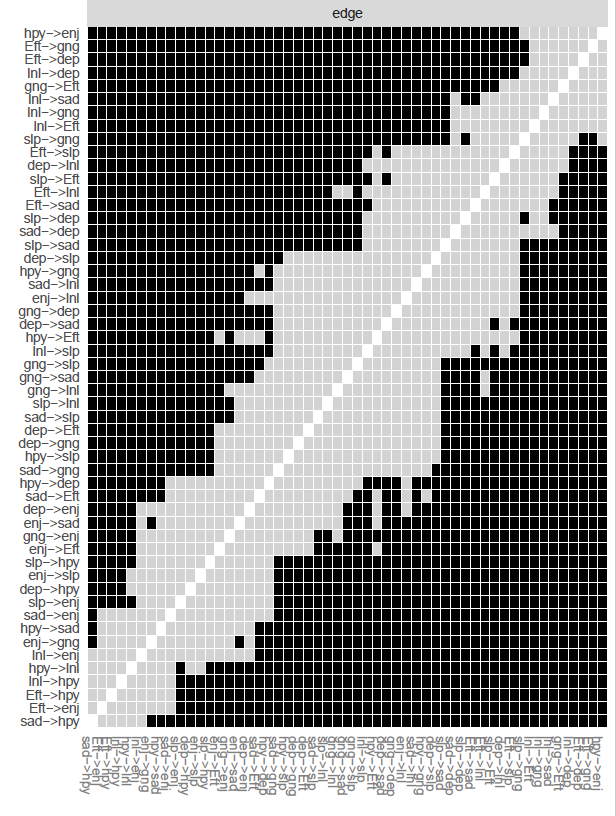


**Figure s22.** Edge weight difference tests for the T3 → T4 network. Black boxes indicate edges that significantly differ from each other (*p* < .05). Gray boxes indicate no differences.

Dep = felt depressed, Eft = everything you did was an effort, slp = restless sleep, hyp = happy, lnl = lonely, enj = enjoyed life, sad = felt sad, gng = could not get going


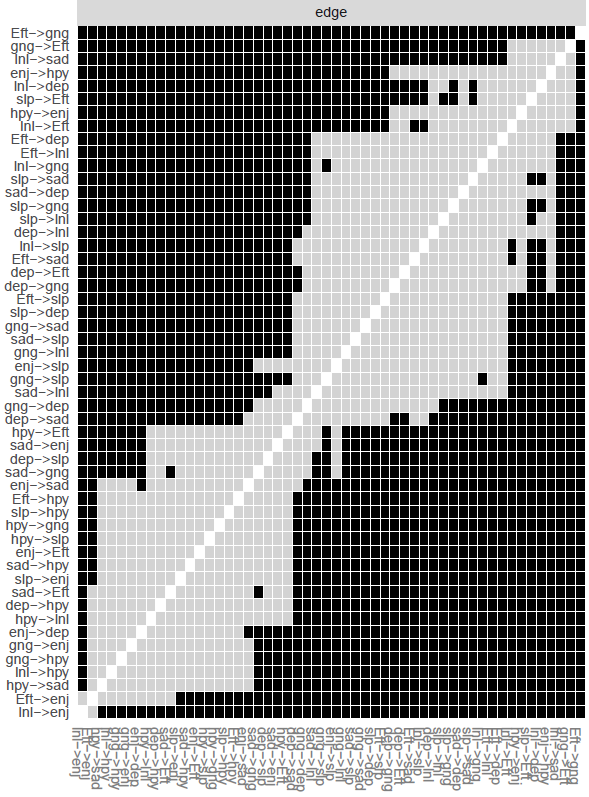


**Figure s23.** Edge weight difference tests for the T4 → T5 network. Black boxes indicate edges that significantly differ from each other (*p* < .05). Gray boxes indicate no differences.

Dep = felt depressed, Eft = everything you did was an effort, slp = restless sleep, hyp = happy, lnl = lonely, enj = enjoyed life, sad = felt sad, gng = could not get going.


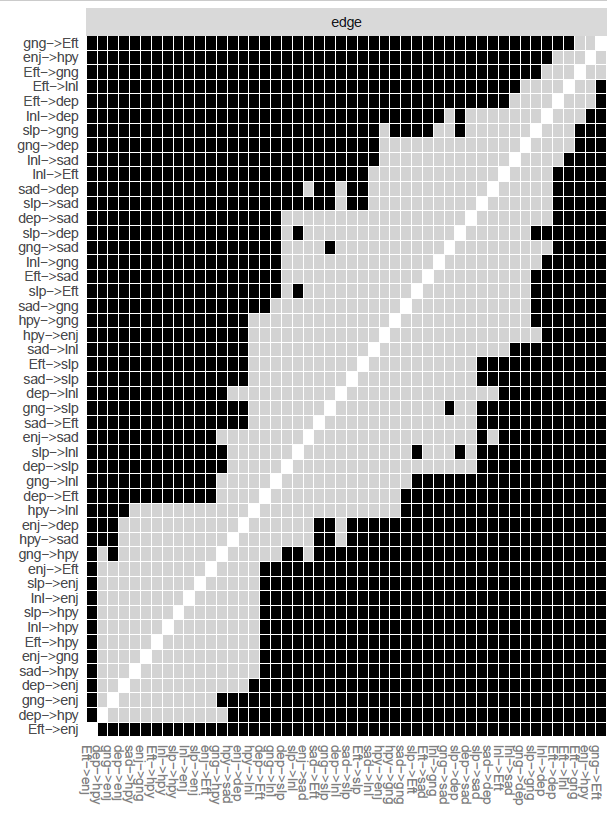


**Figure s24.** Edge weight difference tests for the T5 → T6 network. Black boxes indicate edges that significantly differ from each other (*p* < .05). Gray boxes indicate no differences.

Dep = felt depressed, Eft = everything you did was an effort, slp = restless sleep, hyp = happy, lnl = lonely, enj = enjoyed life, sad = felt sad, gng = could not get going.


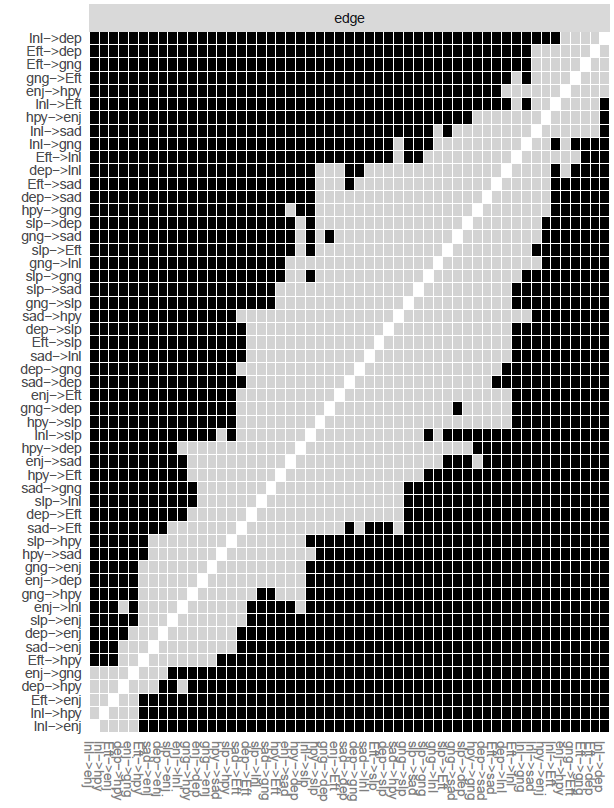


**Figure s25.** Edge weight difference tests for the T6 → T7 network. Black boxes indicate edges that significantly differ from each other (*p* < .05). Gray boxes indicate no differences.

Dep = felt depressed, Eft = everything you did was an effort, slp = restless sleep, hyp = happy, lnl = lonely, enj = enjoyed life, sad = felt sad, gng = could not get going.


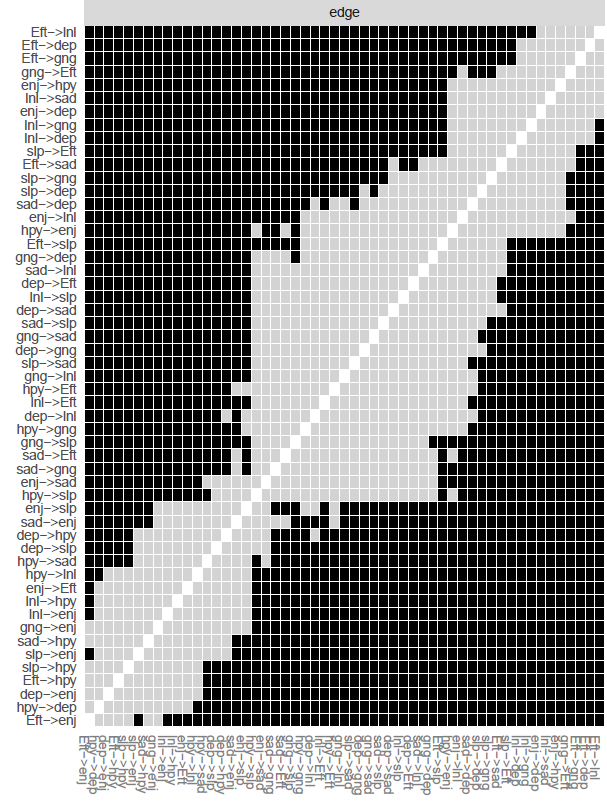


**Figure s26.** Edge weight difference tests for the T7 → T8 network. Black boxes indicate edges that significantly differ from each other (*p* < .05). Gray boxes indicate no differences.

Dep = felt depressed, Eft = everything you did was an effort, slp = restless sleep, hyp = happy, lnl = lonely, enj = enjoyed life, sad = felt sad, gng = could not get going.


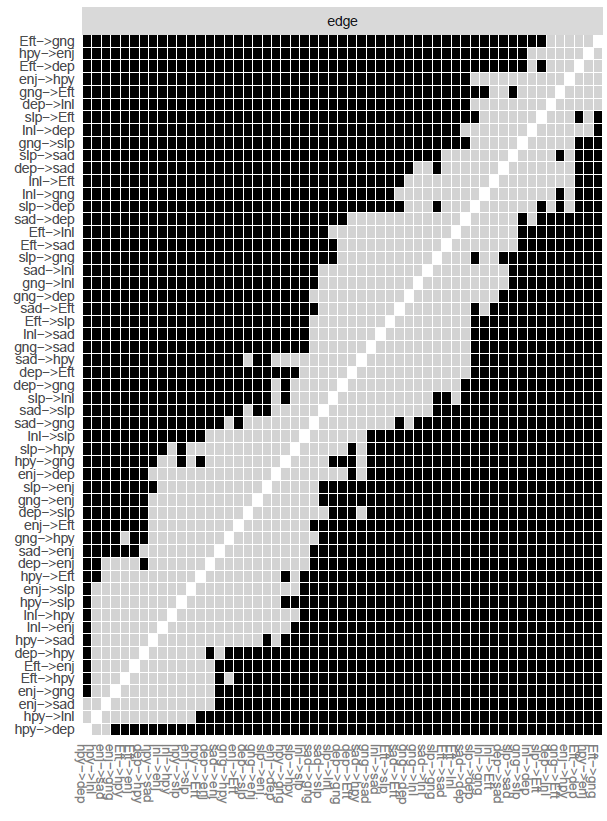


**Figure s27.** Edge weight difference tests for the T8 → T9 network. Black boxes indicate edges that significantly differ from each other (*p* < .05). Gray boxes indicate no differences.

Dep = felt depressed, Eft = everything you did was an effort, slp = restless sleep, hyp = happy, lnl = lonely, enj = enjoyed life, sad = felt sad, gng = could not get going.


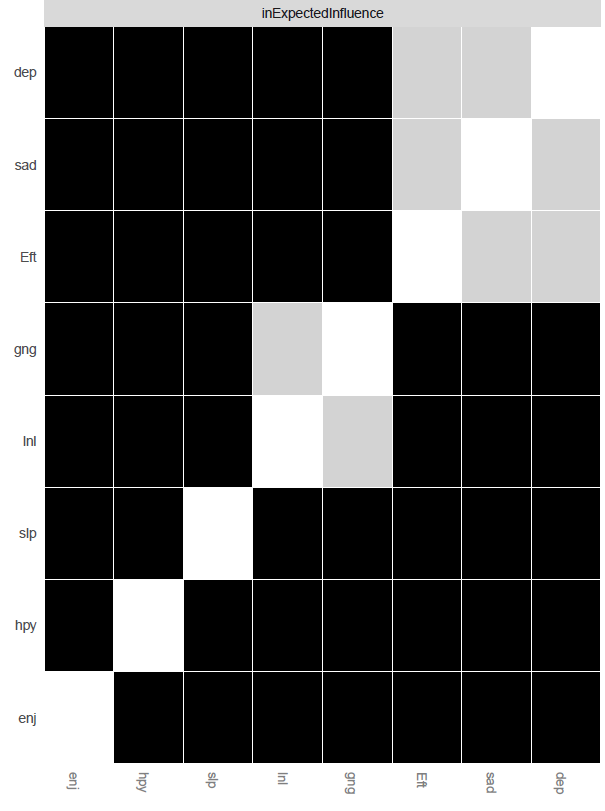


**Figure s28.** In-expected-influence difference tests for the T1 → T2 network. Black boxes indicate edges that significantly differ from each other (*p* < .05). Gray boxes indicate no differences.

Dep = felt depressed, Eft = everything you did was an effort, slp = restless sleep, hyp = happy, lnl = lonely, enj = enjoyed life, sad = felt sad, gng = could not get going


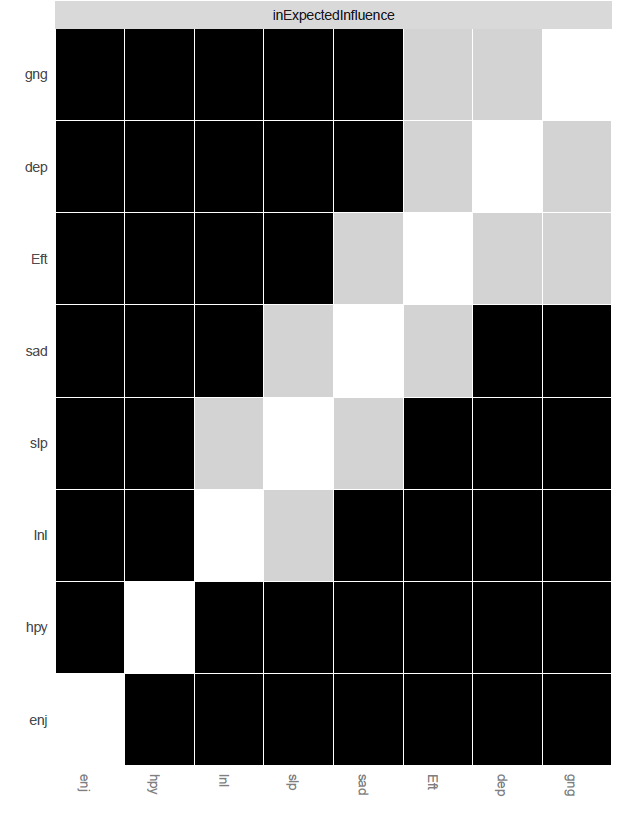


**Figure s29.** In-expected-influence difference tests for the T2 → T3 network. Black boxes indicate edges that significantly differ from each other (*p* < .05). Gray boxes indicate no differences.

Dep = felt depressed, Eft = everything you did was an effort, slp = restless sleep, hyp = happy, lnl = lonely, enj = enjoyed life, sad = felt sad, gng = could not get going.


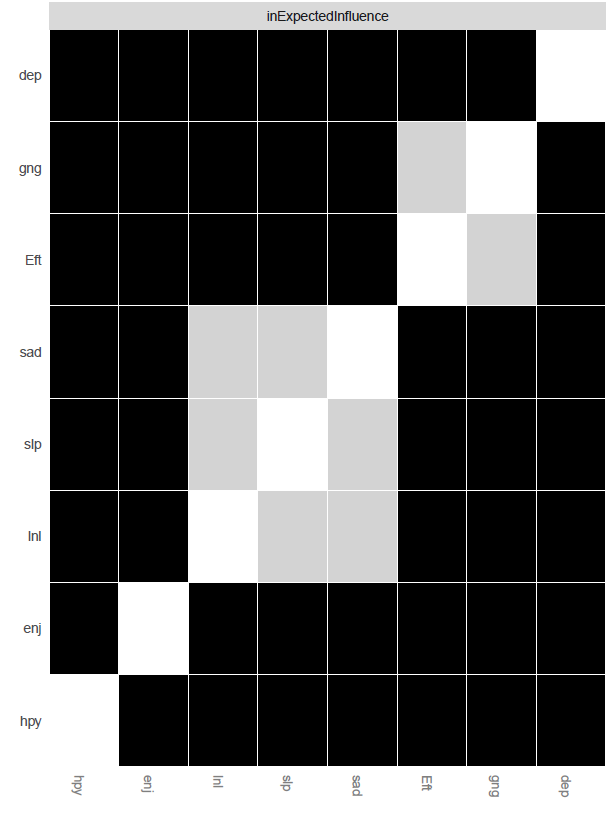


**Figure s30.** In-expected-influence difference tests for the T3 → T4 network. Black boxes indicate edges that significantly differ from each other (*p* < .05). Gray boxes indicate no differences.

Dep = felt depressed, Eft = everything you did was an effort, slp = restless sleep, hyp = happy, lnl = lonely, enj = enjoyed life, sad = felt sad, gng = could not get going.


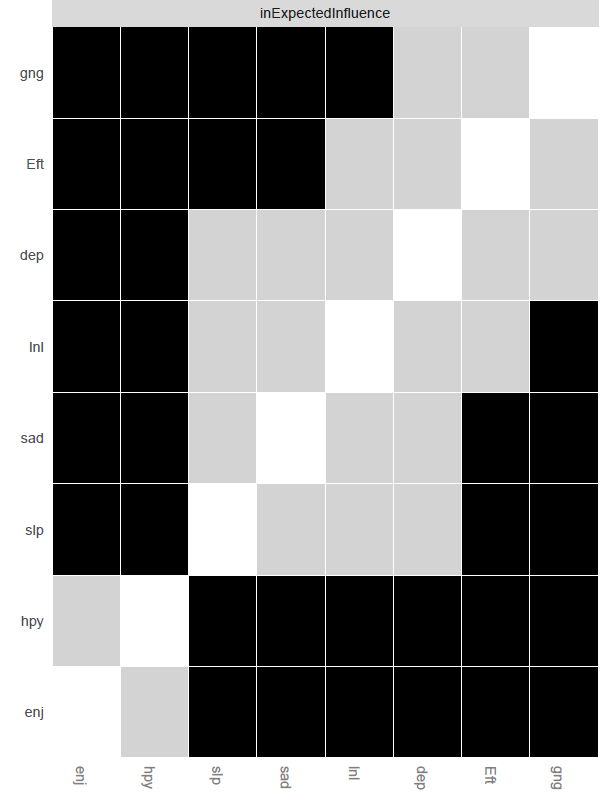


**Figure s31.** In-expected-influence difference tests for the T4 → T5 network. Black boxes indicate edges that significantly differ from each other (*p* < .05). Gray boxes indicate no differences.

Dep = felt depressed, Eft = everything you did was an effort, slp = restless sleep, hyp = happy, lnl = lonely, enj = enjoyed life, sad = felt sad, gng = could not get going.


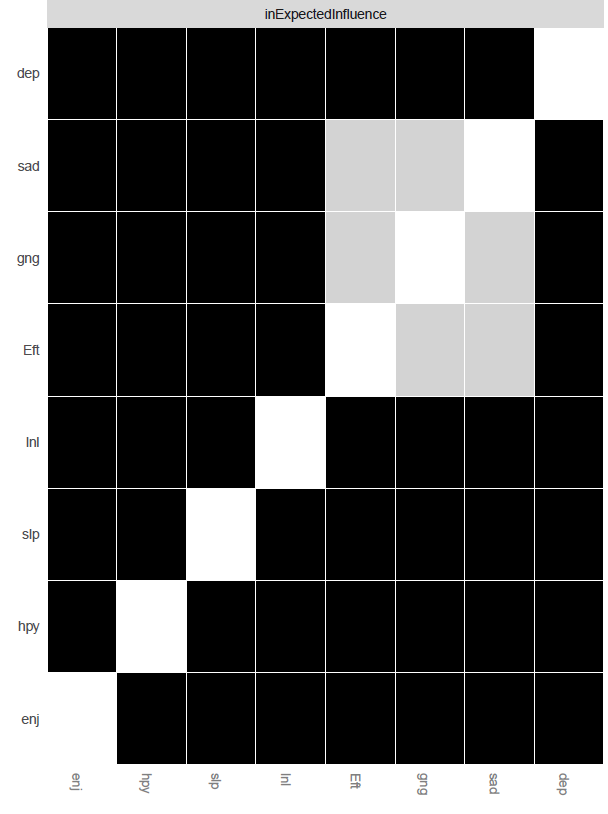


**Figure s32.** In-expected-influence difference tests for the T5 → T6 network. Black boxes indicate edges that significantly differ from each other (*p* < .05). Gray boxes indicate no differences.

Dep = felt depressed, Eft = everything you did was an effort, slp = restless sleep, hyp = happy, lnl = lonely, enj = enjoyed life, sad = felt sad, gng = could not get going.


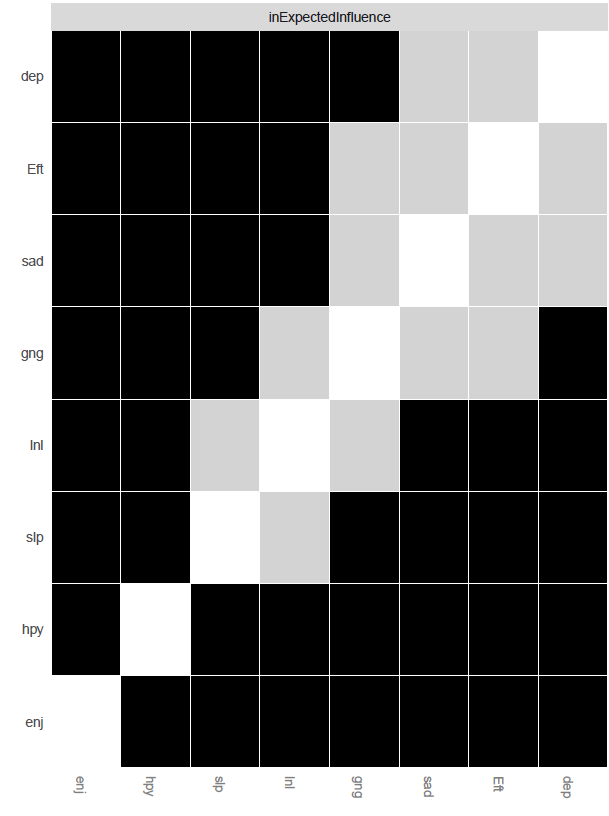


**Figure s33.** In-expected-influence difference tests for the T6 → T7 network. Black boxes indicate edges that significantly differ from each other (*p* < .05). Gray boxes indicate no differences.

Dep = felt depressed, Eft = everything you did was an effort, slp = restless sleep, hyp = happy, lnl = lonely, enj = enjoyed life, sad = felt sad, gng = could not get going.


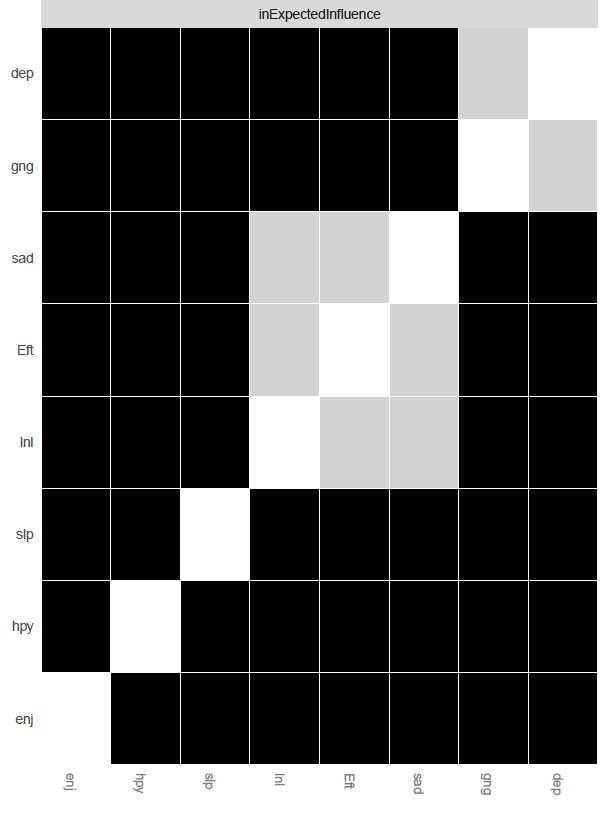


**Figure s34.** In-expected-influence difference tests for the T7 → T8 network. Black boxes indicate edges that significantly differ from each other (*p* < .05). Gray boxes indicate no differences.

Dep = felt depressed, Eft = everything you did was an effort, slp = restless sleep, hyp = happy, lnl = lonely, enj = enjoyed life, sad = felt sad, gng = could not get going.


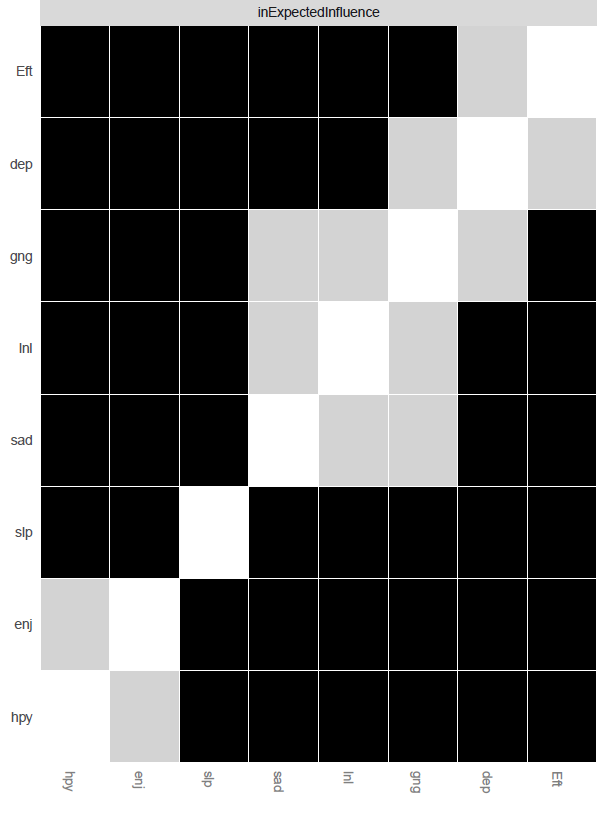


**Figure s35.** In-expected-influence difference tests for the T8 → T9 network. Black boxes indicate edges that significantly differ from each other (*p* < .05). Gray boxes indicate no differences.

Dep = felt depressed, Eft = everything you did was an effort, slp = restless sleep, hyp = happy, lnl = lonely, enj = enjoyed life, sad = felt sad, gng = could not get going.


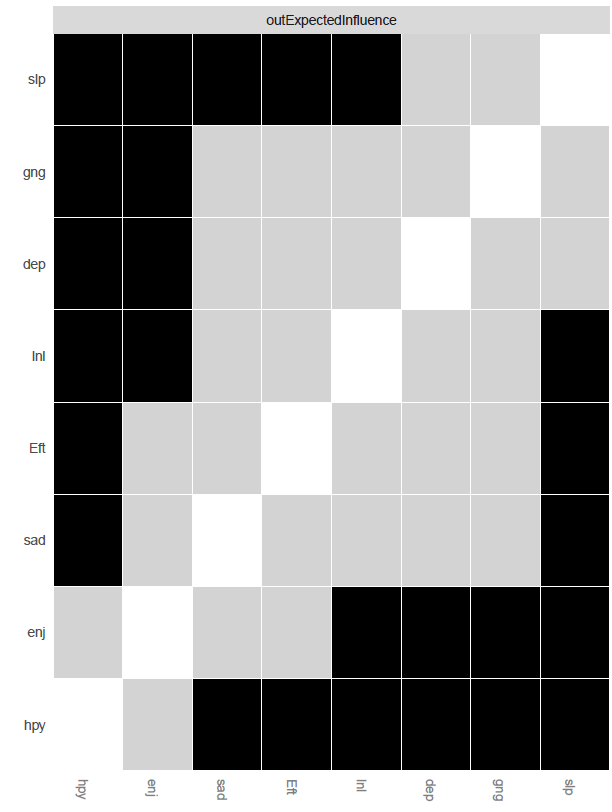


**Figure s36.** Out-expected-influence difference tests for the T1 → T2 network. Black boxes indicate edges that significantly differ from each other (*p* < .05). Gray boxes indicate no differences.

Dep = felt depressed, Eft = everything you did was an effort, slp = restless sleep, hyp = happy, lnl = lonely, enj = enjoyed life, sad = felt sad, gng = could not get going.


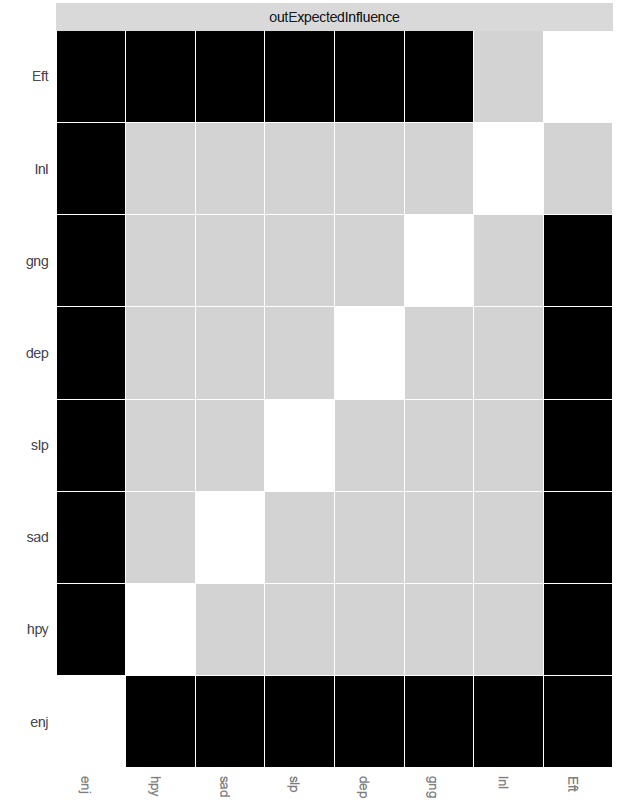


**Figure s37.** Out-expected-influence difference tests for the T2 → T3 network. Black boxes indicate edges that significantly differ from each other (*p* < .05). Gray boxes indicate no differences.

Dep = felt depressed, Eft = everything you did was an effort, slp = restless sleep, hyp = happy, lnl = lonely, enj = enjoyed life, sad = felt sad, gng = could not get going.


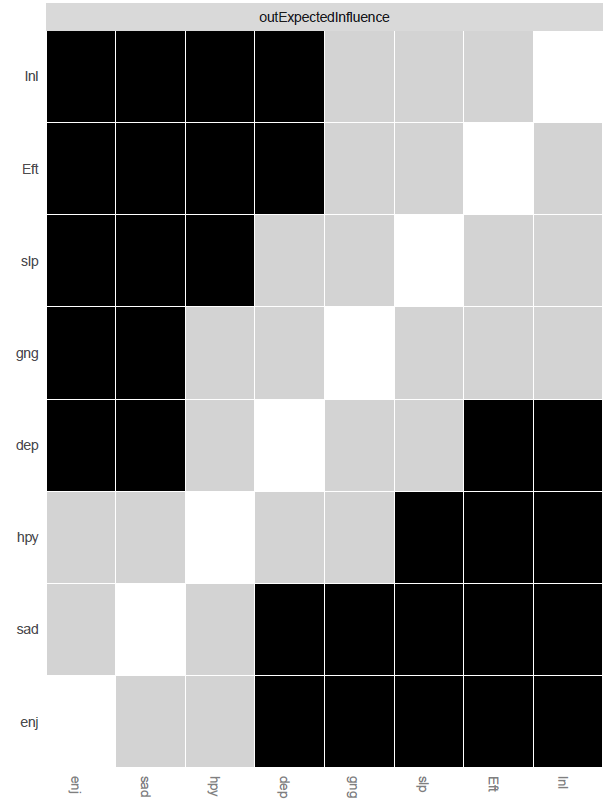
**Figure s38.** Out-expected-influence difference tests for the T3 → T4 network. Black boxes indicate edges that significantly differ from each other (*p* < .05). Gray boxes indicate no differences.

Dep = felt depressed, Eft = everything you did was an effort, slp = restless sleep, hyp = happy, lnl = lonely, enj = enjoyed life, sad = felt sad, gng = could not get going.


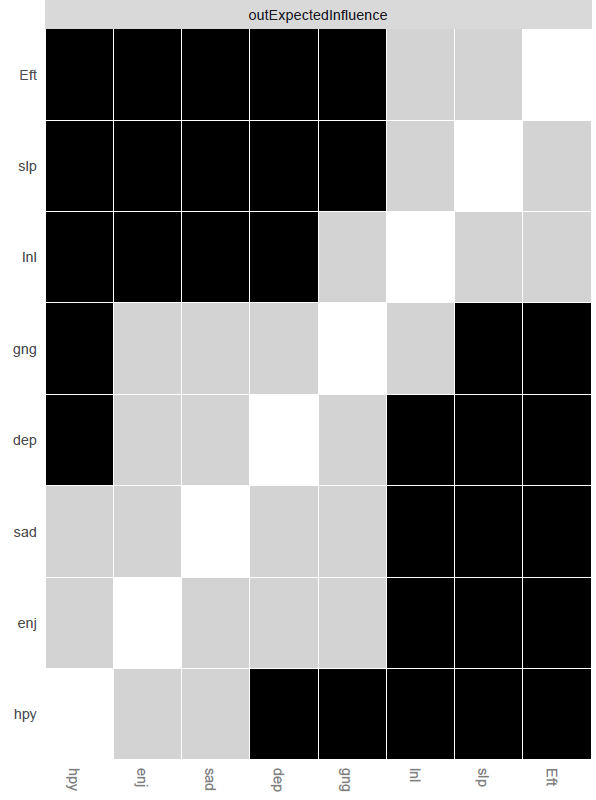


**Figure s39.** Out-expected-influence difference tests for the T4 → T5 network. Black boxes indicate edges that significantly differ from each other (*p* < .05). Gray boxes indicate no differences.

Dep = felt depressed, Eft = everything you did was an effort, slp = restless sleep, hyp = happy, lnl = lonely, enj = enjoyed life, sad = felt sad, gng = could not get going.


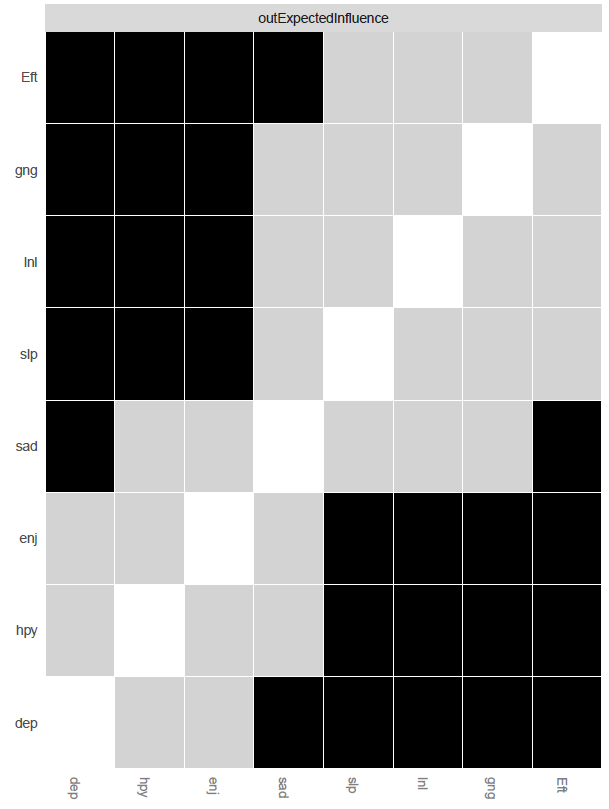


**Figure s40.** Out-expected-influence difference tests for the T5 → T6 network. Black boxes indicate edges that significantly differ from each other (*p* < .05). Gray boxes indicate no differences.

Dep = felt depressed, Eft = everything you did was an effort, slp = restless sleep, hyp = happy, lnl = lonely, enj = enjoyed life, sad = felt sad, gng = could not get going.


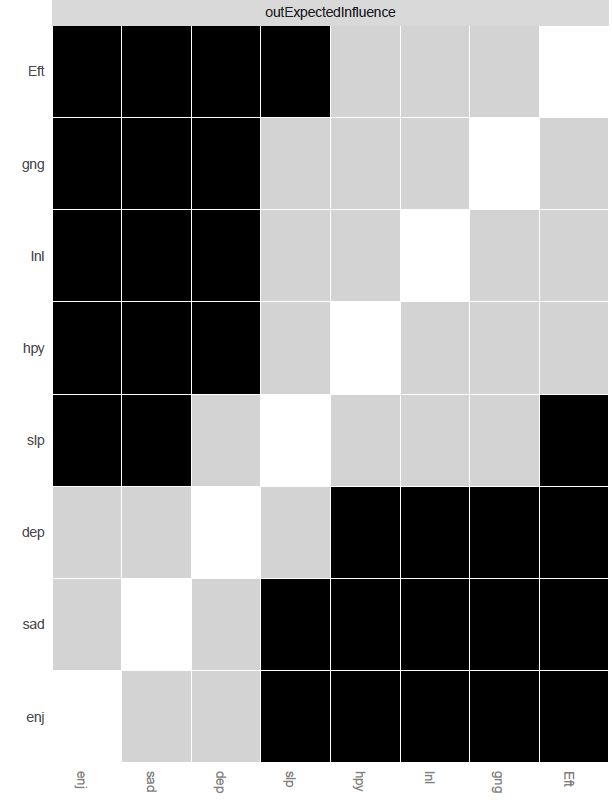


**Figure s41.** Out-expected-influence difference tests for the T6 → T7 network. Black boxes indicate edges that significantly differ from each other (*p* < .05). Gray boxes indicate no differences.

Dep = felt depressed, Eft = everything you did was an effort, slp = restless sleep, hyp = happy, lnl = lonely, enj = enjoyed life, sad = felt sad, gng = could not get going.


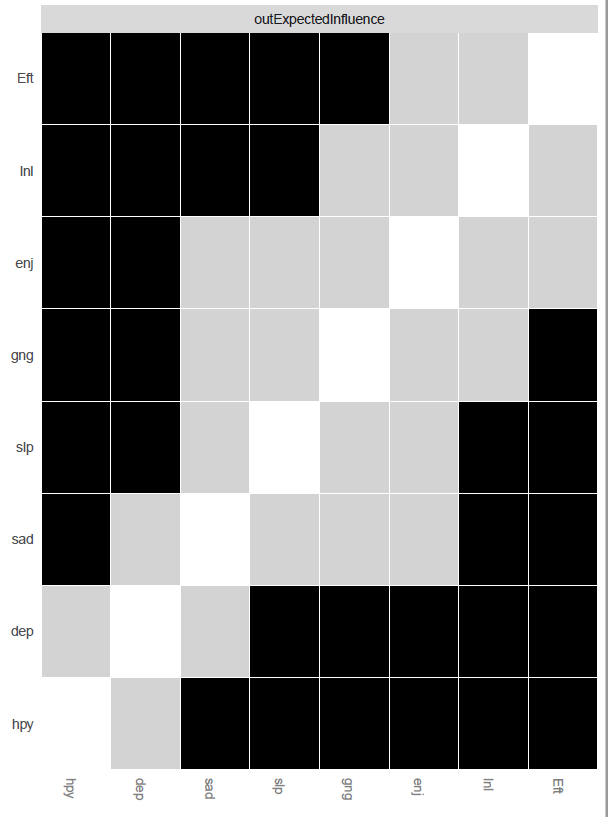


**Figure s42.** Out-expected-influence difference tests for the T7 → T8 network. Black boxes indicate edges that significantly differ from each other (*p* < .05). Gray boxes indicate no differences.

Dep = felt depressed, Eft = everything you did was an effort, slp = restless sleep, hyp = happy, lnl = lonely, enj = enjoyed life, sad = felt sad, gng = could not get going.


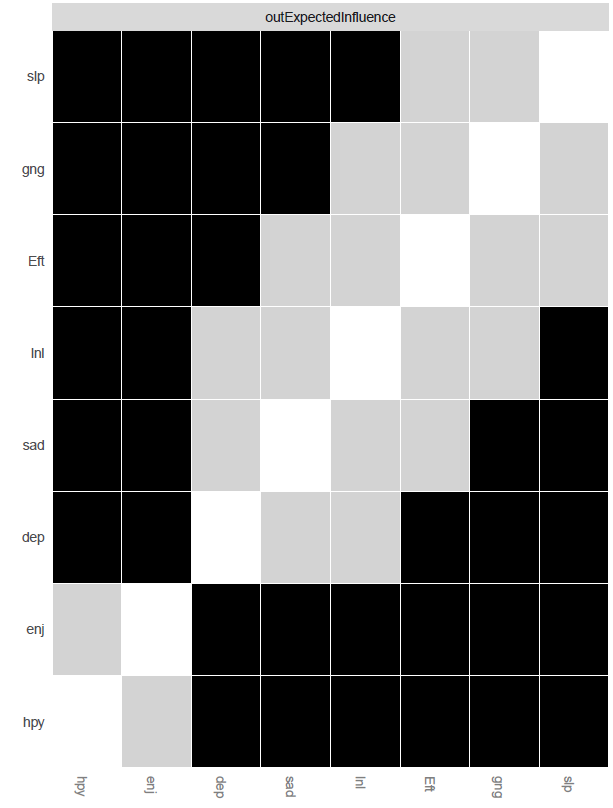


**Figure s43.** Out-expected-influence difference tests for the T8 → T9 network. Black boxes indicate edges that significantly differ from each other (*p* < .05). Gray boxes indicate no differences.

Dep = felt depressed, Eft = everything you did was an effort, slp = restless sleep, hyp = happy, lnl = lonely, enj = enjoyed life, sad = felt sad, gng = could not get going.
